# Deciphering hippocampal place codes in weak theta rhythms

**DOI:** 10.1038/s41467-026-69438-5

**Published:** 2026-02-13

**Authors:** Gautam Agarwal, Seiji Akera, Brian Lustig, Eva Pastalkova, Albert K. Lee, Friedrich T. Sommer

**Affiliations:** 1 Department of Natural Sciences, Pitzer College, Claremont, CA USA; 2 Department of Natural Sciences, Scripps College, Claremont, CA USA; 3 Pitzer College, Claremont, CA USA; 4 Howard Hughes Medical Institute, Beth Israel Deaconess Medical Center, Boston, MA USA; 5 Janelia Research Campus, Howard Hughes Medical Institute, Ashburn, VA USA; 6 Summit Psychology, PLLC, Private Practice, Frisco, CO USA; 7 Helen Wills Neuroscience Institute, UC Berkeley, Berkeley, CA USA; 8 Neuromorphic Computing Lab, Intel Corporation, Santa Clara, CA USA

**Keywords:** Neural decoding, Hippocampus, Dynamical systems

## Abstract

Local field potentials (LFPs) reflect coordination among neural populations, yet their exact relationship to neural computation remains unknown. One exception is the theta rhythm of the rodent hippocampus, which organizes sequential firing among place cells, enabling spike timing to track the animal’s path through its environment. But when the animal stops, the theta rhythm becomes irregular, which is assumed to disrupt its ability to carry spatial information. Here we challenge this assumption by developing an artificial neural network that discovers position-tuned theta rhythms (pThetas) from LFPs even in the absence of strong theta oscillations. Using recordings from male rats, we provide evidence that pTheta is distinct from the dominant theta rhythm, while reflecting rhythmic coordination among place cell populations. Our work suggests that weak and intermittent oscillations, as seen in many brain regions and species, can convey information commensurate with population spike codes when decoded using information-based rather than variance-based principles.

## Abstract (web summary)

Whether weak brain rhythms carry information about ongoing behavior remains unclear. Here, the authors develop a neural network that finds subtle motifs in irregular brain rhythms arising from neural populations to read out where a rat is in its environment.

## Introduction

Recording from an electrode in the brain reveals local field potentials (LFPs) that reflect the coordinated activity of many thousands of neurons. While oscillations in the LFP are a widespread phenomenon^1,2^, their role in brain function remains debated^3^. Proponents point to their computational potential^4–6^, while others question if they are merely epiphenomenal^7^.

The theta rhythm of the rat hippocampus is an extensively studied brain oscillation. Hippocampal neurons called place cells fire when a rat occupies specific locations of its environment, known as the cells’ place fields. When the animal traverses a place field, the cell fires rhythmic bursts at progressively earlier phases of the ongoing theta rhythm, a phenomenon known as phase precession^8^. A consequence of phase precession is that, within a single theta cycle, place cells fire sequentially according to the order in which their place fields are traversed (see Fig. 2 of ref. ^9^). Indeed, the theta rhythm has been found to organize the sequential spiking of neurons across the larger hippocampal-parahippocampal spatial navigation system^10,11^ and beyond^12^. When the animal stops moving, however, theta oscillations become weak and irregular^13^, in principle compromising the inter-regional spike-phase code.

In previous work, we showed that anatomically distributed patterns in the theta band of the LFP, encode the rat’s current location^14^. However, our approach hinged on using a strong theta rhythm as a reference, or carrier, for reading out LFP phase patterns. This limits its generalizability, given that the strength of theta varies across behavioral conditions^13^ and species^15–17^. Here, we ask whether positional information can still be recovered from theta rhythms when they become weak and irregular. One possibility is that a weak theta rhythm cannot sustain coherent activity among place-coding cells, resulting in noisy LFPs incapable of carrying positional information. Instead, we find that position information remains present in LFPs even when theta is weak, but that information is best recovered by detecting place-selective LFP patterns at theta frequency (subsequently termed ‘place theta’ or ‘pθ’) without using the dominant theta rhythm (subsequently termed ‘θ’) as a reference. While a dominant brain rhythm might appear to organize a rhythmic neural code, it is not always best for reading out the information present in co-occurring rhythms that overlap in space, time, and frequency.

All told, even when they are weak, low-frequency LFP rhythms can rival the spatial precision of spike-based codes that demand far greater resources to resolve^18,19^. This matters because a neural signal’s capacity to encode behavior, in part, provides evidence of its importance in neural computation^20^. Low-frequency mesoscopic rhythms may therefore reflect a fundamental level of communication underlying brain function^21^.

## Results

### Carrier-based decoding is less reliable during immobility

**Fig. 1:**
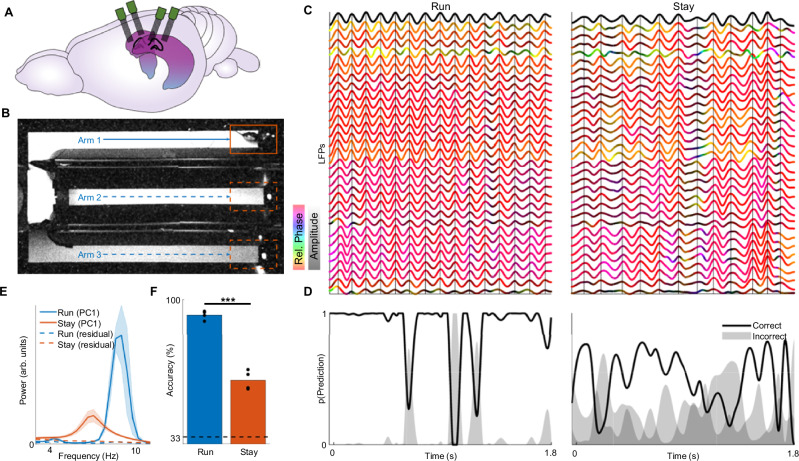
Carrier-based decoding of recorded LFPs during periods of strong and weak theta. **A** Neural activity was recorded from up to 4 64-electrode arrays implanted bilaterally in dorsal CA1. Panel adapted from Tang, W. Hippocampus and Prefrontal Cortex in Rat’s Brain. Zenodo. 10.5281/zenodo.3925923 under a CC BY license: (https://creativecommons.org/licenses/by/4.0/)^69^. **B** Rats were trained to sequentially visit ports in a 3-arm maze to receive water reward, allowing us to sample local field potentials (LFPs) during running (blue) and staying (red). **C** Theta-band LFPs recorded during running (left) and staying (right), respectively showing strong and weak theta rhythms. The top black line indicates θ, the first principal component of the multi-channel LFP. The lines below depict LFPs measured at individual electrodes and are color-coded based on their instantaneous phase relative to θ (yellow - leading, purple - lagging) and amplitude (black - low, colored - high). Vertical lines indicate θ peaks. LFPs show greater amplitude and more consistent phase relationships during running than during staying. **D** A linear classifier predicts arm occupancy during running more consistently than during staying. p(Prediction): probability that the rat is in each arm, with correct arm indicated by black line. **E** θ power spectra during running (blue) and staying (red) across over 4 sessions. Hz: Hertz; arb. units: arbitrary units. **F** Carrier-based decoding is more accurate during running than staying across sessions. s: seconds. ***p = 0.00083, 1-tailed paired t-test, *n* = 4 sessions. **E** depicts mean ± s.e.m., *n* = 4 sessions from 3 rats. Source data and statistics are provided as a Source Data file.

We first attempted to decode position information from LFPs under the assumption that position modulates a single theta rhythm, analogous to how information is carried by a radio signal. We recorded LFPs from dorsal hippocampal region CA1 using up to four 64-electrode arrays while rats navigated a 3-arm maze to collect water rewards (Fig. 1A, B). This task structure allowed us to sample periods when the theta rhythm is strong and consistent (i.e., while running down the maze arms, Fig. 1C left), as well as when it is weak and inconsistent (i.e., while staying at the reward ports, Fig. 1C right; Fig. 1E; and Supplementary Fig. 1A). As proposed previously^14^, we 1) transform the time series measured at each electrode into a complex-valued analytic signal with a defined phase and amplitude^22^; 2) use PCA to identify a single dominant component θ that serves as a global reference or carrier signal; 3) demodulate the signal at each electrode by subtracting the global carrier’s phase from the electrode’s local phase. By shifting from absolute to relative phase, demodulation removes the global theta oscillation, revealing multielectrode phase relationships that can be linearly decoded.; and 4) train a linear classifier on the demodulated LFP to predict which maze arm is being occupied by the animal (Fig. 1D). While the rat is running, θ is strong, and the demodulated LFP has a more consistent form that accurately predicts the animal’s position (Fig. 1C, D, left)^14^. By contrast, when the rat stops running, θ weakens, and the demodulated LFP becomes less consistent and less predictive of the animal’s location (Fig. 1C–F). A reduction is also seen when decoding the animal’s location from the firing rates of hippocampal neurons (Suppementary Fig. 1B). Albeit reduced, the place information found in the LFP reflects a genuine place code, rather than confounding variables such as theta power, movement, or ripple events that might vary across space and influence the decoder (Supplementary Fig. 1C–E).

### Carrier-based decoding is susceptible to shared noise

**Fig. 2:**
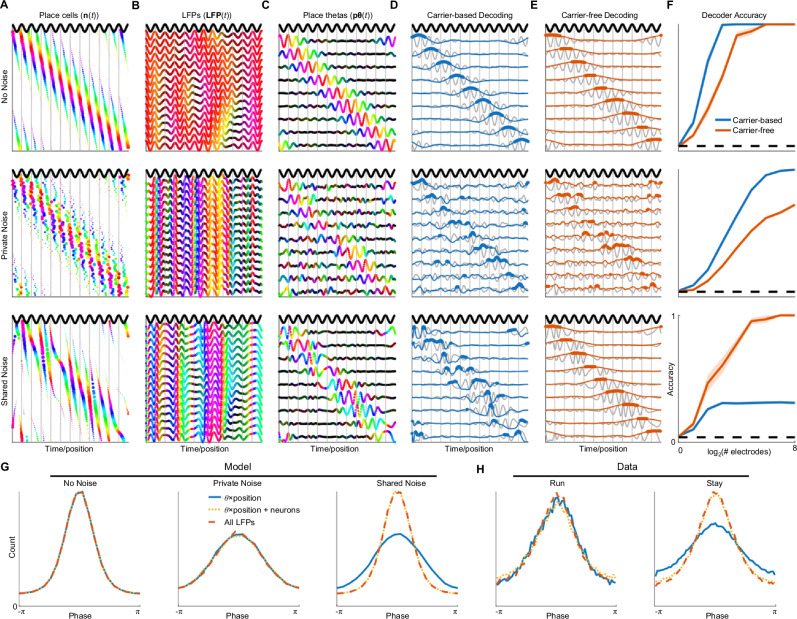
A model reveals how LFP decoding strategy depends on the type of noise. Top row: responses of model neurons contain no noise. Middle row: the phase of each neuron is perturbed independently (private noise). Bottom row - the phase of all neurons is perturbed together (shared noise). **A** Place cell responses for a single traversal of a linear environment. Dot size indicates magnitude of response while color indicates phase of response relative to theta (dark black oscillation). Vertical gray lines indicate theta peaks. **B** Individual electrode responses (LFP(t)) during the same trajectory. **C** Position-varying LFP patterns in (**B**) can be linearly transformed to yield place-tuned theta (pθ) oscillations. In **B**, **C** data is complex-valued, with color indicating relative phase and intensity representing amplitude (as in Fig. 1C). **D** Carrier-based decoding involves taking the real projection of the pθs following demodulation by θ (blue lines) and selecting the largest one at each time step (blue dots). **E** Carrier-free decoding involves taking the absolute value of the pθs and selecting the largest one at each time step (red dots). **D**, **E** Gray traces show pθs duplicated from (**C**). **F** Top: Carrier-based decoding is superior when using fewer electrodes because it is more spatially selective than carrier-free decoding Middle: With private noise, carrier-based decoding remains superior. Bottom: With shared noise, carrier-free decoding is superior because it is invariant to pθs’ phase relative to θ. Shaded regions mean ± s.e.m. of 10 simulated sessions. **G**, **H** Each neuron’s activity was predicted using theta and position alone (blue); using theta, position, and activity of other neurons (yellow); or using all theta-band LFPs (red). Better predictions lead to narrower distributions. **G** Predicted phase of model neuron’s activity when there is no noise (left), private noise (middle), or shared noise (right). Only for the case where there is shared noise, the prediction is improved by including ongoing network activity. **H** Histograms of real CA1 place cells’ spiking as a function of their predicted phase during run (left) and stay (right) periods. Only during stay periods, the prediction is improved by including spiking or LFP activity.

To examine why LFP decoding precision is reduced during staying, we tested a simple model of theta LFP generation (Supplementary Fig. 2). The model consists of a large population of place cells whose place fields tile the environment uniformly (Fig. 2A, top). While the model uses single, localized place fields for simplicity, the outcomes described in this section generalize to cases where cells have multiple, regularly or irregularly spaced place fields (e.g., see simulation in ref. ^14^), as found in circuits that may contribute to place information in CA1 LFPs^23,24^. Without loss of generality, we refer to these in the model as place cells. The responses of these cells are phase-locked to a theta carrier in a position-dependent manner, simulating phase precession^8,25,26^. The LFP at different electrodes is modeled as a linear superposition of the neural responses (Supplementary Fig. 2). The resulting simulated LFPs exhibited slight place-dependent modulations in amplitude and phase that are due to inhomogeneities in electrodes’ sampling of place cell activity and that resemble those found experimentally (Fig. 1C). We can then use linear regression to derive a collection of decoders that detect the multielectrode pattern unique to each position along the track. Together, we refer to the rhythmic LFP components carrying position information as pθ (Fig. 2C, top). We can estimate the animal’s position as the one corresponding to the most active pθ. Because the decoders produce complex-valued outputs, pθ activation can be assessed in different ways. One approach is carrier-based decoding, in which the real component of the demodulated pθ activations is compared (Fig. 2D, top). This approach is sensitive to the phase of each pθ relative to the theta carrier. A second approach is carrier-free decoding, which treats pθs as autonomous rhythms that are not read out using an extrinsic carrier. Mathematically, this is achieved by comparing the magnitudes of pθs (Fig. 2E, top). For the noiseless LFP model, carrier-based decoding is superior to carrier-free because the LFPs’ phase relative to the theta carrier contains information about position (Fig. 2C–F, top), an expected consequence of phase precession. Note that under noiseless conditions, the two methods perform equally well if sufficiently many electrodes are used.

We next modeled noise in the phase coupling between the theta carrier and place cells, either by perturbing neurons’ phase independently (private noise, Fig. 2A–F middle) or together (shared noise, Fig. 2A–F bottom). Private noise changes the phase relationships between neurons, and consequently, between the LFPs measured at different electrodes. This reduces the performance of both carrier-based and carrier-free methods, both of which rely on the fidelity of the multi-electrode patterns that distinguish different pθs from each other. Shared noise, on the other hand, does not affect the phase offsets between place cells or electrodes, only their phase relative to the carrier. As carrier-based decoding is sensitive to this relative phase, it is compromised by shared noise. In contrast, because carrier-free decoding is insensitive to pθs’ phase relative to the carrier, it is robust to the presence of shared noise (Fig. 2F).

What kind of noise corrupts the decoding of real LFPs during stay periods (Fig. 1F)? Previous work suggests that place cells exhibit shared noise, because their activity is better predicted by models that include the ongoing activity of the surrounding network^27–29^. We investigated this in our model by predicting place cell activity from only the model inputs (θ and position), or by also incorporating the activity of the larger network (i.e., from the activity of other neurons or from the multi-channel LFP). Adding noise to the model led to worse predictions, as indicated by broader distributions of predicted phase (Fig. 2G). However, when the noise was shared, predictions improved by including network activity as a regressor, because the network contains a record of the noise affecting the cell.

Consistent with this, we found that prediction of place cell activity in our recordings improved when we incorporated as regressors the activity of other neurons or LFPs - but only during stay periods, not during running (Fig. 2H). This indicates the presence of shared noise among place cells during immobility, which degrades phase-locking of place cell spikes to a weak θ rhythm but can be corrected for using the ongoing dynamics of the population.

In light of these findings, we devised a method for carrier-free decoding of physiological data to remain robust in the presence of shared noise during periods of weak theta.

### A shallow neural network for carrier-free decoding

Decoding the recording data is challenging because 1) unlike the case of the model used above, we lack direct access to the θ oscillation(s) that organize the place code and 2) mapping oscillatory activity onto a (non-oscillatory) position requires some form of nonlinear transformation. Carrier-based decoding achieves 1) by defining the first principal component of the multi-channel LFP as the θ carrier and 2) by subtracting the phase of the carrier from the phase of each channel’s LFP (demodulation) to remove the oscillatory component and enable linear regression. However, if the place code is not phase-locked to the carrier (as seen in the presence of shared noise), carrier-based decoding is compromised (Fig. 2).

**Fig. 3:**
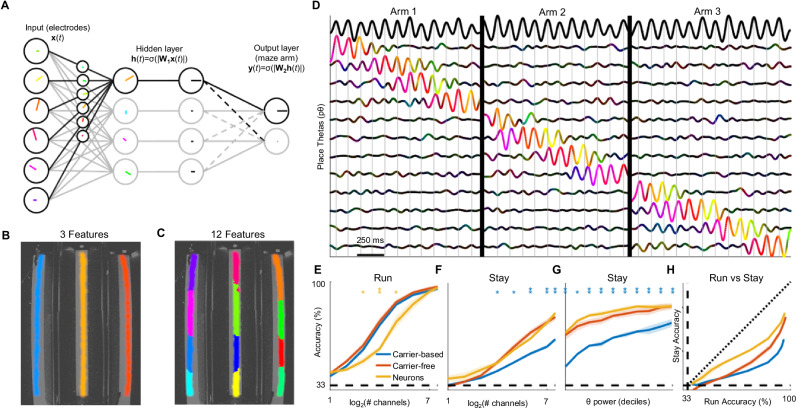
Carrier-free decoding of position from recorded LFPs. **A** TIMBRE architecture, consisting of a hidden layer that takes a complex-valued linear projection of the inputs (${{{\bf{W}}}}_{1}{{\bf{x}}}(t)$). The absolute value of this projection $(|\cdot |)$ is transformed using a softmax operation (σ) on the hidden layer. The hidden layer activations (${{\bf{h}}}(t)$) are projected onto an output layer (${{{\bf{W}}}}_{2}{{\bf{h}}}(t)$) that predicts which maze arm is occupied (${{\bf{y}}}(t)$). **B** When the hidden layer contains 3 nodes, each node learns to represent a single arm. **C**) When the hidden layer contains 12 nodes, each track is tiled uniformly by a subset of the nodes. **D** During running, pθ’s (colored lines, network with 12 hidden nodes) activate sequentially and undergo phase precession relative to θ (top black line). pθ color indicates phase relative to θ and intensity indicates amplitude (as in Fig. 1C). Note that hidden nodes have no intrinsic ordering; we sort them based on the timing of their activation for visualization purposes. Vertical gray lines indicate θ peaks. Vertical black lines separate single runs on each of the 3 maze arms. **E** During running, carrier-based, carrier-free, and spike decoders are similarly effective in predicting the rat’s arm occupancy. Performance is plotted as a function of number of electrodes (for LFP decoders) or neurons (for spike-based decoders). **F** During staying, carrier-free and spike decoders outperform carrier-based decoding. **G** Carrier-based decoding performance shows greater dependency on θ power than carrier-free or spike decoding. Samples are divided into 10 equally sized bins of θ power (deciles). Asterisks indicate significant differences between carrier-free and carrier-based (blue) or spike (yellow) decoders (*p < .05, **p < .01; *n* = 4 sessions). **H** Decoders’ performance during Stay periods relative to Run periods. Lines trace out decoders’ performance across different numbers of electrodes or neurons (see **E**, **F**). Dashed lines indicate chance performance, while the dotted line indicates equivalent performance during ‘run’ and ‘stay’. **E–H** depict mean ± s.e.m., *n *= 4 sessions from 3 rats. Source data and statistics are provided as a Source Data file.

To learn pθs from the data without using a carrier, we designed TIMBRE (Tracking Informative Multivariate Brain Rhythms Efficiently), a complex-valued artificial neural network (ANN)^30^ with three layers (Fig. 3A; Supplementary Fig 3A): 1) A complex-valued input layer that encodes the instantaneous phase and amplitude of theta at each electrode. This representation is equivalent to that used for carrier-based decoding. 2) A hidden layer, in which each node learns a unique place-tuned pattern of phases and amplitudes across input channels (i.e., a pθ component). This is followed by an absolute value operation that discards the phase, and thus the oscillatory component, of the pθ response. Finally, a softmax function is applied, inducing competition among hidden nodes and driving them to learn unique features of the input (Supplementary. Figure 3C). 3) An output layer, each node of which predicts the probability that the rat occupies a particular behavioral state (e.g., maze arm) based on the nodes in the hidden layer that are most active. In summary, TIMBRE’s design allows it to learn behaviorally-tuned oscillatory patterns from physiological data without relying on a carrier.

When applied to physiological recordings, TIMBRE’s hidden layer learns pθ oscillations whose activations uniformly tile the environment; the larger the hidden layer, the finer the tiling (Fig. 3B, C, and Supplementary Fig. 3). Notably, although the network is trained only to predict the maze arm the animal occupies,it spontaneously learns a more precise, fine-grained representation of the rat’s position, a phenomenon known as feature learning^31^. As the rat runs through the maze, pθ components activate sequentially and briefly, exhibiting phase precession relative to the global θ rhythm (Fig. 3D). This sequential activation is made visible by ordering the hidden nodes according to the timing of their activation. During running, the spatially tuned pθ features learned by TIMBRE’s hidden layer closely resemble those learned by independent component analysis (ICA), an unsupervised learning algorithm that identifies sparse, place-specific LFP patterns 13. The hidden layer activations, as visualized by tSNE^32^, trace a clear 1D manifold during running, due to the sequential place cell activation along the linear track (Supplementary Fig. 3E). In contrast, during stay periods, both the manifold structure (Supplementary Fig. 3E) and sequential activation (Supplementary Fig. 3F) disappear, though early and late phases of the stay period show some separation (Supplementary Fig. 3E).

We compared the performance of carrier-based, carrier-free, and spike-rate based decoding as a function of electrodes or neurons used. During running, when θ is strong, carrier-based and carrier-free decoding comparably predict which maze arm is being occupied by the rat (Fig. 3E). In contrast, during stay periods, carrier-free decoding is significantly more accurate at predicting maze arm occupancy (Fig. 3F). Since LFPs arise from neuronal activity, we can use spike rate-based decoding as an upper bound for the amount of information that is recoverable from the neural population. We find that during stay periods, carrier-free decoding largely closes the performance gap that exists between carrier-based decoding and spike decoding (Fig. 3F, G), suggesting that it is effectively recovering the information available in the underlying neural population. This improvement is especially notable when θ power is weak (Fig. 3G). Comparing decoders’ performance between run and stay periods, we see that carrier-free decoding is particularly effective at recovering information from weak oscillations as the number of electrodes increases (Fig. 3F, H). For classifying position, TIMBRE outperforms two dimensionality reduction methods, CSP and ICA (Supplementary Fig. 3D), both of which are commonly used to identify informative oscillations in multi-channel EEG and LFP recordings^14,33,34^.

Periods of immobility are associated with sharp-wave ripple (SWR) events, high-frequency oscillations during which place cells replay trajectories through the environment^35^. Because of their distinct temporal profile and their encoding of remote locations, SWRs would be expected to disrupt theta-based coding of the animal’s current location. We find that decoding performance dips briefly, but consistently stays above chance, around SWR onset (Supplementary Fig. 3G). Because SWRs were rare (0.36 ± 0.04 per second across 3 sessions) and their effects brief, they did not significantly disrupt the encoding of local position by pθs.

### pTheta and theta reflect distinct network components

**Fig. 4:**
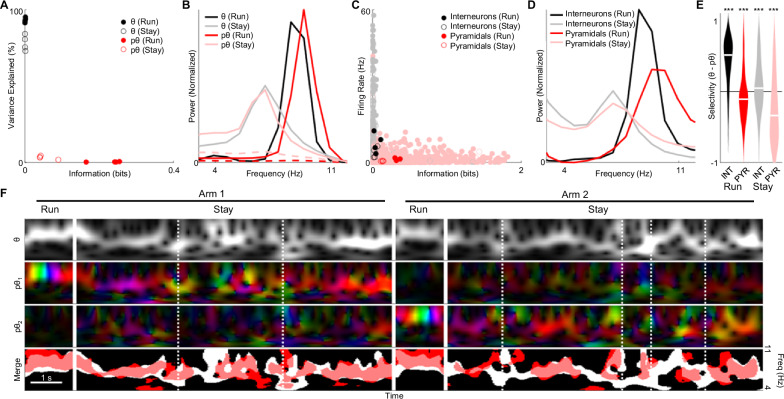
Physiological properties of two theta rhythms. **A** θ accounts for most of the variance of the multi-channel LFP (93% run, 78% stay), but carries no information about animal location. pθ explains little of the multi-channel LFP’s variance (.41% run, 3% stay), but carries more information about animal location. Individual dots indicate variance and information during running (filled) and staying (empty) for 4 sessions. **B** During running, pθ oscillates at a higher frequency than θ due to phase precession. During staying, the peak frequencies of pθ and θ overlap. **C** Interneurons exhibit higher firing rates but are minimally modulated by position. Pyramidal cells exhibit lower firing rates but are strongly modulated by position; Hz: Hertz. **D** During running, pyramidal cells show higher peak frequency than interneurons. During staying, the peak frequencies of the two cell types overlap. **E** Interneuron activity is better predicted by θ, while pyramidal cell activity is better predicted by pθ. ***p < .001, **p <0.01 that the mean selectivity of each class of neurons is 0. *n* = 489/444 (INT/PYR run); 363/181 (INT/PYR stay) from 3 rats over 4 sessions. Violin plot depicts distribution of selectivity across cells, with white band indicating median. **F** θ (top row) and pθ (2nd + 3rd rows) for two arms. pθ_1_ and pθ_2_ correspond to oscillations that are active in arms 1 and 2 respectively; colors indicate phase relative to θ. During running, the relative phase changes due to phase precession. During staying, the relative phase drifts despite unchanging position. (Bottom row) Regions where θ and pθ power exceed threshold are indicated in white and red respectively. During running, pθ shows a consistently higher frequency than θ; during staying, both oscillations show drift in amplitude and frequency that partially overlap. Dotted lines indicate SWR events. Scale bar: 1 second. For **B**, **D** red solid (dashed) lines indicate pθ and place cell spectra for trials in which the rat was in the signals’ preferred (non-preferred) arm; Freq. (Hz): Frequency in Hertz. Source data and statistics are provided as a Source Data file.

We next compare pθ with θ, the classical high-variance oscillatory component that can be identified using PCA and was used here as a carrier. When we reconstruct the multi-electrode LFPs using both θ and pθ, we find that θ explains >80% of the observed variance, while pθ accounts for <3% of the signal. In contrast, only pθ carries information about the animal’s position (Fig. 4A). During running, pθ oscillates at a higher frequency than θ; no such difference is seen during stillness (Fig. 4B)^36^. The differences between θ and pθ parallel those observed between interneurons and pyramidal cells in the hippocampus: the former have higher firing rates that are relatively consistent across the maze, while the latter are strongly position-modulated, remaining silent across much of the environment (Fig. 4C). Furthermore, pyramidal cells oscillate at a higher frequency than interneurons during running (Fig. 4D)^37^. On average, we find that interneuron activity is better predicted by θ, while pyramidal cell activity is better predicted by pθ both during running and staying (Fig. 4E), even after accounting for the differences in place tuning among these constituents. The difference in peak frequency and θ/pθ selectivity (Fig. 4D, E) is not explained by cell-type differences in cluster isolation or firing rate (Supplementary Fig. 4D).

Observing θ and pθ on single trials reveals their complex and somewhat distinct dynamics. As the rat runs across the track, the higher frequency of pθ results in phase precession relative to θ. During staying, θ and pθ show a gradual drift in their relative phase and partial overlap in their power across frequency and time (Fig. 4F).

### Carrier-free decoding in the open field

**Fig. 5:**
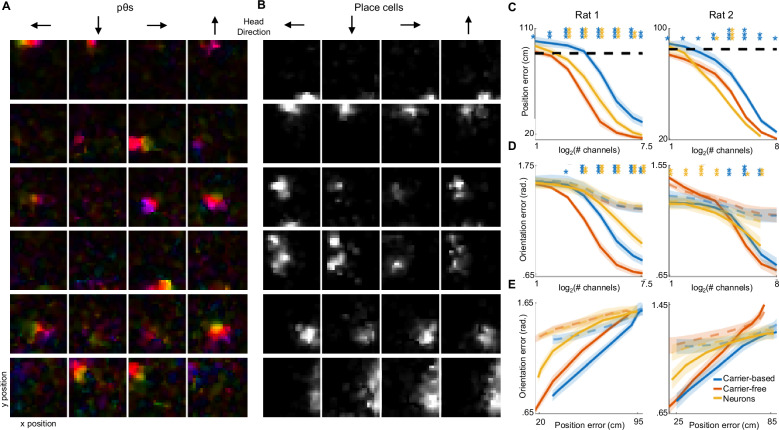
Decoding recorded LFPs during open field foraging. **A** Responses of 6 hidden nodes of TIMBRE as a function of rat position and orientation in an open field (152.4 cm × 152.4 cm). Colors represent the phase of each pθ relative to θ. **B** Responses of 6 pyramidal cells as a function of position and orientation in the open field. **C–E** Mean error of carrier-based, carrier-free, and spike decoders in the open field for two rats. **C** Position error as a function of # channels or neurons; **D** Orientation error as a function of # channels or neurons; **E** Orientation error as a function of position error. For **C** black dashed line indicates chance-level performance; for **D** and **E** colored lines indicate chance-level performance of orientation decoding achievable by position decoders, accounting for position-dependent orientation preference of each rat. Shaded regions show mean ± s.e.m. of test accuracy, *n* = 10 (8) folds for rat 1 (2). For rat 2, two folds were excluded due to distributional shift (see “methods”). cm: centimeters; rad.: radians. Source data and statistics are provided as a Source Data file.

We evaluated TIMBRE’s ability to extract information about position from theta oscillations during foraging in the open field. Because behavior is less constrained during foraging than in the maze, while also spanning a broad range of θ power (Supplementary Fig. 5C), it offers a test of the robustness of carrier-free decoding. When trained using weak supervision (Supplementary Fig. 5A), we find that the pθ components learned by TIMBRE’s hidden layer are localized both in x-y position and heading direction (Fig. 5A). In contrast, individual place cells, while localized in position, often lack orientation selectivity (Fig. 5B). Each learned pθ component shows phase precession relative to θ (Fig. 5A).

We compared carrier-based, carrier-free, and spike-based decoding of position and orientation in the open field (Fig. 5C–E, and Supplementary Fig 5D, E). All decoding methods improved as the number of electrodes or temporal smoothing increased, with carrier-free decoding outperforming carrier-based decoding. The spike-based decoder could decode head direction^19^; however, the LFP decoders did so more effectively (Fig. 5D), consistent with the direction selectivity intrinsic to pθ (Fig. 5A). Decoding also improved at faster velocities for all decoders. Carrier-free decoding showed more stable performance across velocities than carrier-based decoding (Supplementary Fig. 5C). TIMBRE performed comparably to a convolutional neural network known as DeepInsight^19^, although the latter relies on higher sampling rates to incorporate spiking activity, derives little information from LFPs below 150 Hz, is substantially more complex, and takes >500x longer to train.

## Discussion

Interpreting LFPs is challenging due to their complex relationship to neural activity and poor spatio-temporal localization^38^. One exception is the hippocampal theta rhythm, which appears to serve as a canonical brain clock coordinating sequential spiking in place cells^10^. We previously showed that theta phase could provide a reference for reading out phase-amplitude patterns in LFPs^14^. However, under behavioral conditions where theta weakens, it becomes unreliable as a reference. Here, we show that phase-amplitude patterns can directly be read out from LFPs without recourse to a theta clock. This finding suggests that place-encoding neural networks maintain a phase-position code, at least partially independent of the classically observed theta rhythm^39^.

During running, place cell spikes exhibit phase precession relative to theta. We find analogous phase precession in pθ (Figs. 3D, 4B, 4F, 5A). Because pθ and θ are tightly coordinated during running, carrier-free and carrier-based models predict position equally well (Fig. 1C–E left, 2E, 3B). Consequently, during running, we find no evidence that place cell assemblies oscillate independent of theta. Carrier-free decoding with TIMBRE reveals place-tuned rhythmic components that trace a 1D manifold (Supplementary Fig. 3), as expected from coherent place cell populations. We find different hidden layer sizes to reveal different degrees of place field localization (Supplementary Fig. 3B), reminiscent of the multi-scale nature of the place code^40^.

When locomotion stops, a weak and irregular Type II theta is observed (Fig. 1F; Fig. 4B, F)^13^. In this condition, carrier-based decoding suffers (Fig. 1G). One possible explanation for this drop is that when theta is weak, it can no longer synchronize place cells and consequently fails to generate informative patterns in the LFP. Alternatively, place cells could generate coordinated activity independent of theta. Consistent with the second explanation, a carrier-free model better predicts position across a range of θ power, similar to spike-based decoding (Fig. 3F-G). Unlike during running, during stay periods decoding improves by using multiple hidden nodes to represent each location. This suggests that a drop in θ power leads to more variability in place cell population activity, and by extension, in position-tuned LFP patterns. While the time-averaged power spectra of pθ and θ overlap (Fig. 4B), the two rhythms show distinct time-varying activity (Fig. 4F). During both run and stay periods pθ and θ predict pyramidal cell and interneuron activity, respectively. The association between pθ and pyramidal cells extends beyond their place selectivity, suggesting that pθ is reporting noise correlations (shared noise) among place cells. This is further supported by the observation that the spike timing of place cells is more accurately predicted by models that incorporate the larger network’s ongoing activity as reported by spikes or LFPs (Supplementary Fig. 2B, C).

The open field provides a third behavioral context for examining pθ. Unlike the linear track, in the open field place cells show localized place fields that are invariant to the rat’s direction of motion (Fig. 5B). While only trained to classify position, TIMBRE’s hidden layer reveals pθs that are selective for both position and orientation and that phase-precess in the direction of motion (Fig. 5A). We find that neural spiking contains some information about head direction, consistent with the discovery of interneurons whose firing rates are modulated by orientation24. However, we find LFP-based head-direction decoding to be superior (Fig. 5C–E), pointing to a source of information that extends beyond firing rates alone. Given that place cells exhibit phase precession in the open field^41^, we would expect these cells to activate in an orientation-dependent order. Consequently, movement in each orientation would evoke distinct phase patterns in the LFP. The orientation selectivity of pθ thus provides further evidence that it reports sequential activity within place cell assemblies.

It is important to note that these statistical associations do not imply that pθ exclusively reports CA1 place cell activity. Instead, pθ likely reports the activity of the larger hippocampal-entorhinal circuit involved in estimating the location of the animal.

A further contribution of this work is TIMBRE, a simple and interpretable neural network that extracts oscillations that are predictive of behavior. TIMBRE achieves carrier-free decoding by learning oscillatory patterns whose amplitudes vary in a behavior-dependent manner. We found that using a softmax transfer function encourages competition among nodes in the hidden layer so that they learn features that tile the behavioral space. When using carrier-free and spike-based decoders as baselines, TIMBRE appears to be more successful in decoding position in the maze than in the open field. One potential explanation for this difference is that the maze involves a more stereotyped behavior in which each position is heavily sampled. In contrast, foraging in the open field invokes a more diverse behavioral repertoire, presumably evoking a wider array of LFP patterns. At the same time, the open field is less thoroughly sampled, limiting the training data for each hidden node. Finally, while our recordings had at most 255 channels, we see evidence that decoding will continue to improve with more electrodes, both in the maze (Fig. 3F) and the open field (Fig. 5C, D).

TIMBRE complements many existing computational approaches that have been developed to decode and interpret multi-channel brain wave data. Like complex-valued ICA (cICA)^42^, it learns a complex-valued linear projection of the data that maximizes a particular objective. When applied to the theta rhythm during running, these algorithms produce similar results (compare Figs. 3C–4A of ref. ^14^). Because cICA is fully unsupervised, its learned features may include artifacts unrelated to brain function^43^. In contrast, TIMBRE aims to discover a feature space that is maximally informative about a behavior of interest. Another related approach is Common Spatial Patterns (CSP), which is used to identify oscillatory subspaces whose variance changes maximally across conditions^44^. We find CSP to be inadequate for decoding and interpreting theta LFPs (Supplementary Fig 3D), suggesting its orthogonality and variance constraints are unsuitable for revealing low-variance multi-electrode oscillatory patterns that occupy a manifold (Supplementary Fig. 3E). Another parallel is found in shallow convolutional networks that have been developed to detect behavior-encoding waveforms from EEG data^45,46^. In particular, EEGNet reduces the number of parameters by enforcing spatio-temporal separability within its learned features. However, TIMBRE has the advantage of being able to detect phase offsets across electrodes, invariant of timescale. Because place cells preserve phase relationships despite changes in theta frequency^47^, TIMBRE can detect pθ even as its center frequency shifts, as is seen during periods of stillness (Fig. 4F). We therefore believe that TIMBRE is ideal for multi-electrode recordings in brain regions where neurons are phase-coupled and respond to a measurable behavior or condition.

This work raises several questions for future research. First, what are the neural underpinnings of pθ and θ, respectively? One possibility is that these correspond to the two components in the dual oscillator model^8^, with θ reflecting pacemaker input from the medial septum and pθ potentially signaling the collective activity of place cells and/or inputs from grid cells within the entorhinal cortex^11^ (Supplementary Fig. 6). While it remains possible that both rhythms arise from a single network^36^, we believe that the distinction between pθ and θ is meaningful, in that they selectively correlate with pyramidal cells and interneurons respectively (Fig. 4E). Recordings including multiple regions, such as medial septum, CA3, and entorhinal cortex, all of which could contribute to CA1 LFPs^23,24,48^, would be invaluable in identifying the extent of the circuitry from which pθ and θ arise. Second, what are the functional implications of the greater variability observed in theta during stay periods? On the one hand, this variability could result from a low-fidelity representation of self-position due to reduced navigational demands during stay periods. On the other hand, the variability in phase coupling we observe (Fig. 4F) could result from a more varied behavioral repertoire during staying, potentially indicating shifting contributions of memory encoding and retrieval^49^. In conjunction with mechanistic and behavioral interventions, TIMBRE could help address such questions by providing a mesoscopic view of neural codes.

Over the past century, neuronal spikes have emerged as the essential currency of neural computation^50^. However, a dedicated minority has argued that mesoscopic electric fields, available in the lower frequency range of physiological recordings, reveal additional mechanisms underlying neural function^1,21^. The longstanding counterargument has been that mesoscopic signals offer only impoverished representations, averaging away the rich dynamics of single neurons. Yet recent technologies have revealed that large neural populations often occupy surprisingly low-dimensional subspaces of activity^51^. Thus, local field potentials (LFPs), despite aggregating over the responses of individual neurons, could in principle fully reflect the computations occurring at the population level. Previously, this possibility was confirmed in the hippocampus by decoding from robust LFP oscillations the same behavioral information carried by neural spiking. However, most brain circuits exhibit weak or irregular rhythmic activity embedded within dominant broadband fluctuations, complicating the recovery of information from LFPs based on variance alone.^52^. Here, we demonstrate that even when neural activity is only weakly phase-locked to the dominant rhythm in a region, neurons remain coordinated through shared variability. Because both this shared variability and neural spatial representations are inherently low-dimensional, we were able to read out the animal’s position from these weakly rhythmic LFP signals. Our findings complement proposals^5,53,54^ that rhythms organize and define robust and efficient spike-based computing and communication. Thus, mesoscopic rhythmic patterns, while seemingly harder to interpret than spike trains, can be as revealing of the brain’s computational logic.

## Methods

### Subjects

Adult male Long-Evans rats were used for experiments. All procedures were performed according to the Janelia Research Campus Institutional Animal Care and Use Committee guidelines on animal welfare.

### Three arm delayed sequence task

The Three Arm Delayed Sequence task (TADS) requires the rat to visit maze arms for reward in the following order: left, center, right, and center. The rat starts each trial in the delay area and has to run in a wheel in a fixed direction continuously for eight seconds over a set speed threshold to activate all three maze arm doors to open. The 2.13m-long maze (Fig. 1B) was designed by Eva Pastalkova and Tanya Tabachnik in the Instrument Design & Fabrication facility at Janelia Research Campus. The design and operation of the maze is described in ref. ^55^.

### Open field foraging task

Water-restricted animals were placed on a large rectangular platform (152.4 cm × 152.4 cm) with walls^56^. To motivate exploration, small balls of wet food (rolled from food pellets soaked in water) were sprinkled onto the surface by an experimenter approximately every 2–4 min. Sessions were 43 minutes (rat 1) and 32 minutes (rat 2).

### Recording neural activity

We performed chronic extracellular recording in freely behaving rats performing the spatial memory task. Chronic extracellular recording of neural activity was performed using silicon probes from Neuronexus (Buzsaki 64 and BuzsakiSP 64 probes). 3 d printed ring, external faraday cage, and nanodrives were from Ronal Tools (http://www.ronal.com/). Ring was designed by Eva Pastalkova and Yingxue Wang. Implant procedure was by Eva Pastalkova. Faraday cage was designed by Brian and Andy Lustig.

Neural signals and tracking of position was carried out using the Amplipex recording system (http://www.amplipex.com/). Importantly the Amplipex system is integrated with Intan multiplexing headstages (http://www.intantech.com/index.html) which permit recording from large numbers of channels over a thin light cable to minimize interference with behavior in the freely moving rats. Commutators for recording were either custom from Dragonfly (www.dragonflyinc.com) or Doric Lenses (ERJ 12 HDMI-B2). Neural activity was recorded at 20 kHz.

Each rat had 4 64-electrode shanks implanted straddling the superficial to deep pyramidal layers of dorsal CA1. For the three-arm delayed sequence task, data is shown from 4 sessions collected using 3 rats. For the rat that produced two of these sessions, one shank was excluded due to poor signal quality (resulting in signals from 64*3 = 192 electrodes). For the open field foraging task, data is shown from 2 sessions collected using 2 rats. For rat 2, one shank was excluded due to poor signal quality (resulting in signals from 192 electrodes).

Neural recordings were stored as.dat files. Behavioral location within the maze was recorded by tracking of LEDs on the rat’s implant at 30 Hz using a webcam integrated with the Amplipex system. A sync pulse from the maze behavioral control system was recorded on the Amplipex system in order to guarantee accurate syncing of behavioral information and neural activity recorded on Amplipex. For LFPs, recordings were downsampled to 1250 Hz and saved as.lfp files.

### Spike sorting and interneuron classification

The NDManager and Klusters suite was used for analysis as well as MATLAB. Spike detection was performed using high pass filtered signals and threshold crossings above the mean + 1.5 SD. Then spike waveforms were stored and sorted using the KlustaKwik suite. After automatic sorting, clustering was further refined manually using Klusters^57^. To separate interneurons and pyramidal cells, we identify 6 features in each neuron’s action potential waveform (Extended Fig. 4A^58^): a1) Left peak height a2) right peak height b) left peak width c) right peak width d) center peak height f) center peak width. Because a1, a2, and d were heavily right-skewed distributions, we took the log of each feature. We then standardized the data by z-scoring each feature. Finally we performed k-means clustering to identify 2 clusters. The projection of these features onto 2 principal components was plotted for visualization (Extended Fig. 4B). Clusters were identified as interneuron or pyramidal cells based on their observed features (Extended Fig. 4C). Cluster isolation quality (Extended Fig. 4D) was assessed by comparing within-cluster distance to across-cluster distance^59^. Counts across sessions were as follows:

**Table:** 

Session	Electrodes	Pyramidals	Interneurons
1	255	365	237
2	255	98	85
3	192	305	96
4	192	260	53

### LFP preprocessing

LFP recordings were 1) downsampled from 1250 Hz–25 Hz (using Matlab’s decimate function, which applies a 10 Hz, 8th-order Chebyshev Type I low-pass filter prior to downsampling to prevent aliasing). 2) high-pass filtered (2 Hz, 2nd-order Butterworth filter, zero-phase filtering performed using Matlab’s filtfilt function). 3) Hilbert-transformed to produce a complex-valued signal (using Matlab’s ‘hilbert’ function) that represented the instantaneous amplitude and phase. Additional pre-processing for carrier-based and carrier-free decoding is described in the next 2 paragraphs respectively.

For carrier-based decoding, the first PC (the “carrier” or θ) of the complex-valued LFP was identified using matlab’s svd function. The phase of the carrier was subtracted from the phase of each channel to produce a demodulated LFP

1 $${{\bf{dLFP}}}(t)\,={|}{{\bf{LFP}}}(t)|{e}^{({\varphi }_{{{\bf{LFP}}}(t)}-{\varphi }_{{{\boldsymbol{\theta }}}(t)})i}$$

Finally, the signal was converted from a *T* x *N* complex-valued matrix to a *T* x *2 N* real-valued matrix (i.e., with real and imaginary components concatenated).

For carrier-free decoding, the LFP was whitened by using matlab’s svd function to generate the score matrix **V**, the columns of which (denoted **V**_*i*_) were rescaled using

2 $${{{\bf{V}}}}_{i}=\frac{{{{\bf{V}}}}_{i}}{\sqrt{{{\mathrm{var}}}({{{\bf{V}}}}_{i})+{10}^{-5}\times {\sum }_{i=1}^{N}{{\mathrm{var}}}{{{\bf{V}}}}_{i}}}$$

Analogous to ridge regression, this transformation rescales the data but reduces the contribution of the smallest whitened dimensions, which tend to be noisier.

### LFP analysis in the maze

#### Identifying run and stay periods

Run periods were defined as times when the rat was in one of the three maze arms approaching a reward port. Other periods, including when the rat was running away from the reward port, were discarded as these periods had less consistent running behavior (i.e., with frequent stops) across trials and sessions. Stay periods were defined as times when the rat was positioned near (i.e., within 10 cm of) a reward port for the purpose of consuming water rewards. Unrewarded trials were discarded as these consisted of very brief stay periods. Run and Stay periods were analyzed separately for all subsequent analyses. To determine the velocities in each period (Supplementary Fig. 1), x-y position of the rat was low-pass filtered at 2 Hz. This served to remove jitter in head position arising from noise in position estimation and head undulations, resulting in an estimate of more sustained, directional movements. Speed was computed as the hypotenuse of the change in filtered x-y position.

#### Data selection for cross-validation

Data from each trial was randomly assigned to one of 5 folds, ensuring that data used for model training and testing were separated in time. Since rats traversed the center arm twice as often, and stayed at some reward ports longer than others, data was sub-sampled so that each arm was equally represented for the purpose of model training (i.e., so that the model would not be biased to select the most occupied arm), and such that all 5 folds had equal amounts of training data. 100 random fold assignments were tested, and the assignment that produced the largest amount of training data, given the above subsampling procedure, was chosen.

#### Probabilistic decoding of arm occupancy (Fig. 1D)

L1-regularized logistic regression (liblinear^60^) was used to predict from the demodulated LFP which arm was being occupied by the rat. The cost parameter was set to its default value of 1. The probability of each arm being occupied was determined on a held-out trial using liblinear’s predict function. Decoder performance was compared between run and stay periods using a 1-tailed t-test (*n* = 4 sessions with 3 rats).

#### Carrier-based decoding (Fig. 1F and 3E–G)

An L1-regularized logistic classifier was trained using either run or stay data to predict from the demodulated LFP which maze arm the rat occupied. For running, each track was divided into four sections, for a total of 12 classes. This accounted for the changing place code along the length of the track. The classifier was evaluated on held-out data. Accuracy was defined as the percent of samples whose predicted category (12 for run, 3 for stay) was found in the arm currently occupied by the rat. To evaluate LFP (spike) decoders’ dependency on the number of electrodes (neurons), n channels were randomly sampled and used as inputs to the decoder, where n sampled powers of 2 from 2 to 181 ( ~ 2^7.5). Performance was averaged over 10 repetitions of this random sampling procedure.

#### Carrier-free decoding (TIMBRE)

A complex-valued neural network was implemented in Keras using the complexnn package^30^. The network has one hidden layer, with both the real and imaginary components of the weights of each node constrained such that their L2 norm equals 1. The output of this layer was transformed using the absolute value function, thus discarding the phase, followed by a softmax operation. This operation was found to induce competition between hidden nodes, encouraging them to learn different features. The hidden layer was fed into an output layer with 3 nodes (one for each maze arm) with a softmax operation that estimates the probability of each arm’s occupancy. The network was trained using Adam optimizer, learning rate =0.001, and categorical cross-entropy loss function. Training a network with the combination of cross-entropy loss and softmax has been shown to maximize mutual information between the inputs and the outputs^61^. Training was stopped at 100 epochs, or when the test loss increased (i.e., early stopping), whichever came first (usually the latter condition, in practice). Decoder performance was compared to carrier-based and spike-based decoding using a 2-tailed t-test (Fig. 3E-G; *n* = 4 sessions with 3 rats).

#### Spike-based decoding

A TxN matrix was defined, each entry containing the number of spikes fired by each neuron within a 25-Hz time window. The columns of this matrix were low-pass filtered (3 Hz, 8th-order Butterworth filter, zero-phase filtering performed using Matlab’s filtfilt function). The frequency was chosen so that spike decoding was roughly the same accuracy as carrier-free decoding during running (lower cutoff frequencies resulted in smoother signals that provided better decoding). Each neuron’s response was normalized by its standard deviation calculated within run or stay periods. Logistic regression was then performed analogously to carrier-based decoding, described above. Supplementary Fig. 1B*:* Linear decoding was compared to a Bayesian decoder^62^. Because the latter assumes Poisson counts, neural activity was encoded in a $T\times N$ matrix of spike counts in each time bin. For the Run condition, position along each track was rescaled to the range [0,1] and represented by a 10 × 1 evenly spaced grid of radial basis:

3 $${{{\bf{RBF}}}}_{i}=c{e}^{-\frac{{(x-{x}_{i})}^{2}}{2{\sigma }_{x}^{2}}}$$

where ${x}$ is the animal’s position along the track, ${x}_{i}$ is the RBF’s center position, ${\sigma }_{x}=1/9$ (chosen so that RBF width is concordant with its spacing), and $c$ is a scaling constant such that the RBFs sum to 1. Separate RBFs were used for each of the three tracks for a total of 10 × 3 = 30. For Bayesian decoding, the prior probability of occupancy was defined to be uniform, since the rat’s velocity was consistent across arms and training data was sampled uniformly from the 3 arms. Under a uniform prior and an independent-Poisson model, the likelihood of observing a spike-count vector ${{\bf{n}}}=({n}_{1},\ldots,{n}_{N})$ in a single time bin, given position $x$, is

4 $$P({{\bf{n}}}\,|\, x)\propto \left(i={\prod }_{i=1}^{N}{{f}_{i}\left(x\right)}^{{n}_{i}}\right)\exp \left[-{\sum }_{i=1}^{N}{f}_{i}(x)\right]$$

where ${f}_{i}(x)$ is neuron $i$’s tuning-curve value at position $x$. Because only the most likely position is required and $P(x)$ is uniform, the normalization constant is omitted and the MAP position estimate reduces to ${{{\rm{argmax}}}}_{x}P({{\bf{n}}}| x)$. The linear decoder was trained using ridge regression to predict the RBF representation of position, and the decoded position was taken as the bin with the largest predicted RBF activity. For both decoders, the maze arm was assigned based on the decoded position, and accuracy was defined as the fraction of time bins in which the arm was correctly predicted. For Stay periods, the comparison was analogous but used a single decoding bin per arm.

##### Control analyses for non-place contributions to decoding (Supplementary Fig. 1C–E)

To determine whether TIMBRE’s decoding performance during immobility could be explained by non-spatial correlates of location, we implemented a series of control analyses targeting known behavioral and physiological variables. These included theta power and frequency, x- and y-velocity, ripple events and ripple-band power, reward-related lick behavior, and occupancy of stay region (to capture any signals arising from arrival to or departure from port). Each variable was transformed using a continuous wavelet transform (CWT) to allow the decoder to exploit its temporal structure. CWT was calculated using Matlab’s built-in function, with an analytic Morse wavelet, 2 voices per octave between 2 Hz to 8$\sqrt{2}$ Hz (i.e., spanning the frequencies used for LFP analysis). The amplitude of the CWT was used for decoding. While water delivery at each reward port was tightly controlled using calibrated syringe pumps (ensuring identical reward volumes and flow rates), differences in licking behavior could still arise due to variability in how animals interacted with the ports. Lick detection was available in two of the four sessions. We verified that including or excluding lick variables did not affect the conclusions described below (likely because lick events were correlated with head position, which was already included as a regressor) and thus excluded them from the final analysis for consistency across sessions.

We evaluated seven decoding models in a two-arm classification task:

1-2. Maze arm decoded from the LFP using carrier-based (1) or carrier-free (2) decoding

3. Maze arm decoded from all non-place variables using a linear decoder

4–5. Maze arm decoded from non-place variables reconstructed from the LFP using carrier-based (4) or carrier-free (5) regression

6–7. As in models 4–5, but trained using LFPs from a third maze arm in order to hold the animal’s position constant; the model was then tested on its ability to decode between the remaining two arms

Model 3 tested whether non-place variables contained spatial information. Models 4 and 5 tested whether the LFP encoded these variables in a way that could support indirect place decoding. Models 6 and 7 tested whether mappings from LFP to non-place variables generalized across space. A failure of models 6–7 to decode arm identity indicates that LFP-based decoding performance during immobility cannot be explained by these known non-place variables.

Chance for each model was calculated as percent accuracy for shuffled predictions, ~50% for the two-arm classification task. *n* = 4 sessions, averaged across 3 arm pairs and 5 cross-validation folds. Significance was assessed relative to chance-level performance using a two-tailed paired t-test.

#### pθ rendering

The preprocessed LFP was multiplied by the conjugate of the hidden layer’s weights, producing one pθ oscillation per hidden node. *Spatial rendering* (Fig. 3B, C)*:* the magnitudes of pθ activations were binned by 2-d position within the track and averaged per bin using matlab’s accumarray function. Locations were binned into square bins, such that there were 200 bins along the length of the track. Before rendering, spatial maps were smoothed with a gaussian kernel with $\sigma$ = 2 bins. Each pixel was assigned a unique color corresponding to the most active pθ at that location, with the alpha channel set to the average activation for the pθ. Activations exceeding 1 were set to 1. *Temporal rendering* (Fig. 3D)*:* pθs were sorted by their peak activation within the 3 arms. pθ phases were rotated so that they had 0 average phase lag with respect to θ (as estimated using Matlab’s correlation function). Each pθ response was normalized, dividing by the 95th percentile of its response magnitude. Data from 3 trials, one per arm, were concatenated. Plots show real projection of data, with color hue indicating phase of the demodulated pθ (i.e., pθ phase relative to θ) and intensity indicating response amplitude (with black representing low-amplitude). *Manifold visualization* (Supplementary Fig. 3E): The magnitudes of pθs across 1 session (from TIMBRE with 48 hidden nodes), for held-out data from either Run or Stay periods, were fed into matlab’s built-in tSNE function, using the correlation distance metric. Each arm’s data was color coded using a separate color, with progressively brighter colors assigned to later parts of each trial. Data was 2x downsampled for the longer Stay periods using Matlab’s decimate function, to reduce run time.

#### Comparison to CSP and ICA (Supplementary Fig. 3C-D)

Víctor Martínez-Cagigal’s implementation of CSP^63^ was downloaded from Matlab’s File Exchange. It was modified to handle multiple classes of data by, for each class, solving the generalized eigenvalue problem ${{{\bf{C}}}}_{i}{{\bf{x}}}\,=\,\lambda {{\bf{Cx}}}$, where ${{{\bf{C}}}}_{i}$ is the covariance matrix for complex-valued LFPs recorded when the rat was in arm $i$, whereas ${{\bf{C}}}$ was the covariance matrix for data collected in all arms. The top n eigenvectors, as determined by their eigenvalues, were taken for each arm. For complex-valued ICA^42^, a vectorized version of the ACMNsym matlab function was used and the top n components, as determined by the L2 norms of the components of the mixing matrix, were taken for each arm. For both CSP and ICA, the amplitudes of their activations were used to predict the current arm using L1-regularized logistic regression (Supplementary Fig. 3D), and averaged by position to generate spatial activation profiles (Supplementary Fig. 3C). Decoder performance was compared to TIMBRE using a 1-tailed t-test (Supplementary Fig. 3D; *n* = 4 sessions with 3 rats).

#### Detection of sharp-wave ripples (SWRs) (Supplementary Fig. 3G)

The power in the 150-250 Hz range (using Matlab’s ‘bandpower’ function) was computed and averaged across all electrodes in CA1. SWR events were defined as samples where the z-scored power exceeded 2. This procedure was unable to detect SWR events in 1 of 4 sessions, which was therefore excluded from SWR-related analysis. To plot peri-SWR accuracy, the average accuracy at each time lag relative to a threshold-crossing event was averaged. To determine the rate of SWRs per session, local maxima in the z-scored ripple power exceeding a value of 2 were counted as individual events.

#### Dependence of accuracy on θ power (Fig. 3G)

Each data sample was assigned to one of 10 deciles based on the θ power at each moment. Because SWR events caused a spurious increase in estimated θ power, deciles were defined using relative, rather than absolute, power. Relative θ power was computed as the fraction of the total power in the multi-electrode signal explained by the first principle component.

#### Power spectra (Fig. 4B, D)

The Chronux package (chronux.org^64^) was used to generate power spectra. Specifically, since trials were of unequal length, a short time fourier transform (STFT) was taken on consecutive windows of data within the appropriate trial period (i.e., during run or stay periods) with the taper parameter set to [1/T, T, 1], where T = window size in seconds. The resulting power spectra were averaged across the session. Figure 1*:* SVD was used to produce principal components of the LFP. The spectrum for the first PC was plotted during run and stay, and the spectra of the remaining PCs were added together to generate the power spectrum of the residuals. A T = 1.5 s window was used. Figure 4: For θ, the spectrum of the first PC was taken. For pθ and place cells, the spectrum of each signal was taken separately in each arm. The ‘preferred arm’ for each signal was defined as the arm in which the signal had the largest standard deviation. The other two arms constituted the ‘non-preferred arms’. All spectra (across 3 pθ’s, or all place cells) were averaged together to generate the preferred arm (solid line) or non-preferred arm (dashed line) spectra. For each session, spectra (representing all arms for θ and interneurons, or preferred arms for pθ and place cells) were normalized so that their area summed to 1, prior to averaging across sessions. Spectra for non-preferred arms (for pθ and place cells) were scaled to maintain their power relative to the preferred arm spectra. Error bars were excluded for clarity.

#### Wavelet transform (Fig. 4F)

A period of one trial from each of two arms was selected (1.52 sec for Run and 10 sec for Stay). A continuous wavelet transform was performed on the real component of θ or pθ (derived from TIMBRE trained with 1 hidden node per maze arm) using Matlab function cwt, with default choices for wavelet (the complex-valued Morse wavelet) and bandwidth parameter (60) with 8 voices per octave and frequency range 2 Hz to 8$\sqrt{2}$ Hz. The resulting complex-valued signals were demodulated using the phase of θ, resulting in a zero-phase signal for θ and a slowly-varying phase for pθ (either due to phase precession during running or phase drift during staying). The Matlab function scatteredInterpolant was used to transform the logarithmic frequency axis used by cwt to a linear one. Each signal was normalized by its standard deviation during run or stay periods, across all arms (for θ) or within the active arm (for pθ). For rendering, values of 0.9 or greater were set to maximum intensity, and a threshold of 0.4 was used to define active regions for the merge image (bottom panel).

#### LFP variance explained by θ vs pθ (Fig. 4A)

For either the still or the run periods within each session, linear regression was performed to predict the multi-electrode LFP (bandpass-filtered and Hilbert transformed as described above) from θ and 3 pθs (learned by TIMBRE with 3 hidden nodes). The samples used for training this model were the same as those used to estimate θ and pθ. Using samples from the test set, the variance of the reconstruction was calculated for each of the four regressors (θ and 3 pθs). This variance was normalized by the total variance of the LFP, and the variance explained by the 3 pθs was summed to produce a single number.

There were two important choices that influence our estimate of the fraction of the multi-channel LFP explained by pθ. First, we predicted the LFP jointly from θ and pθ, allowing θ to explain away variance that would otherwise be captured by pθ. We did this because the LFP is so highly correlated, that any random projection of the data would appear to explain a substantial portion of the LFP. Second, we trained TIMBRE with 3, rather than more, nodes. Since pθs are projections of the LFP, larger numbers of pθs tend to overfit the LFP, fitting noise in addition to the position-encoding components of the LFP. Fundamentally, the finding that pθ and θ respectively reflect high-information and high-variance components of the LFP parallels our earlier finding that variance is concentrated in few dimensions but information is distributed across the full dimensionality of the LFP (Fig. 2C of^14^).

#### Neuronal activity explained by θ vs pθ (Fig. 4E)

For each neuron, spikes were binned at 25 Hz sampling rate. The resulting vector was 1) low-pass filtered (10 Hz, 8th-order Butterworth filter, zero-phase filtering performed using Matlab’s ‘filtfilt’ function); 2) high-pass filtered (2 Hz, 2nd-order Butterworth filter using filtfilt); 3) Hilbert-transformed to produce a complex-valued signal (using Matlab’s hilbert function). This produced a signal analogous to the preprocessed LFP (see above). For each session, TIMBRE was trained using 3 hidden nodes, resulting in one pθ that activated in each maze arm. Data was separated by arm and behavioral condition, resulting in six conditions per neuron. For each condition, the response of each active neuron (defined as those with firing rate greater than 2 Hz within the condition) were predicted using a linear model with θ and the active pθ as regressors. Each neuron’s selectivity ${s}$ was calculated using the learned weights ${w}_{\theta }$ and *w*_*pθ*_ as

5 $$S=\frac{|{w}_{\theta }|-|{w}_{p\theta }|}{|{w}_{\theta }|+|{w}_{p\theta }|}$$

The distribution of s was rendered using H. Hoffmann’s violin.m function, avaialable through Matlab Central. Because the regression was done separately for data from each arm, and because only one pθ was active in each arm, the selectivity of pyramidal cells for pθ was not due to their both being tuned to position (because pθ did not show position tuning within the condition). A two-tailed t-test was performed on the selectivity of each cell group (interneurons and pyramidal cells) within each behavioral condition (run vs stay) with the null hypothesis that the selectivity was not significantly different from 0.

#### Position information in LFPs and Neurons (Fig. 4A and C)

We calculated the information carried by LFPs or spike trains about position using the formula

6 $${Info}={\sum }_{i=1}^{3}{{{\bf{P}}}}_{i}\left({{{\bf{R}}}}_{i}/{{\bf{R}}}\right){\log }_{2}({{{\bf{R}}}}_{i}/{{\bf{R}}})$$

where ${{{\bf{P}}}}_{i}$ is the probability of occupying maze arm $i$, ${{{\bf{R}}}}_{i}$ is the average response (firing rate for neurons, amplitude for θ and pθ) in maze arm $i$, and ${{\bf{R}}}$ is the average response. For very sparse signals, this formula reports artificially high values^65^; therefore, the information was calculated after randomly shifting the time series of maze arm occupancy relative to the time series of LFP/neuron responses, and averaged over 100 time-shifts. This randomized estimate was subtracted from the originally estimated information.

### LFP analysis in the Open Field

#### Visualizing position-orientation tuning (Fig. 4A and B)

TIMBRE was trained with a hidden layer of 256 nodes to classify the rat’s position within the left or right half of the open field (Supplementary Fig. 5). Compared to the linear track, the pθs were less strongly position-tuned. We therefore visualized the pθs in the open field after applying the softmax function to their amplitudes. This had the effect of suppressing the activity of hidden nodes in the locations where they were weakly activated. Finally, we demodulated the pθ’s by subtracting θ’s phase from the phase of each pθ. We then discretized the rats’ position into 24 bins along the *x*- and *y*-axis and into 4 orientations. We averaged the pθ or neuronal responses within each bin and applied a Gaussian spatial filter of radius = 1 bin for the responses within each of the 4 orientations. For visualizing neural place fields, we discarded the phases (leaving just the amplitudes) because spiking had a less consistent phase relative to θ, leading to noisier place fields when phase was kept. Example pθs (Fig. 4A) and neuronal (Fig. 4B) place-orientation fields were manually selected to highlight fields that were in the middle, rather than the edge, of the field. This is because the rat spent significant time running around the edges, and neuronal responses on the edge resembled those on the linear track, giving less insight into the nature of open-field responses.

#### Position and orientation decoding (Fig. 4C)

Data from the session was divided into 10 consecutive, equally sized folds for the purposes of cross-validation. Position was rescaled so that it covered the range [0,1]. Position and orientation were represented by a 10 × 10 × 10 evenly spaced grid of radial basis functions using the formula

7 $${{{\bf{RBF}}}}_{{ijk}}=c{e}^{-\left(\frac{{(x-{x}_{i})}^{2}+{(y-{y}_{j})}^{2}}{2{\sigma }_{{xy}}^{2}}+\frac{{(\min \left(\right|\varphi -{\varphi }_{k}|,\,2\pi -|\varphi -{\varphi }_{k}|)}^{2}}{2{\sigma }_{\varphi }^{2}}\right)}$$

where $x$,$y$, and $\varphi$ represent the animal’s 2 d position and orientation; ${x}_{i},{y}_{j},{and}$ ${\varphi }_{k}$ represent the RBF’s center in position and orientation; ${\sigma }_{{xy}}=1/9$ and ${\sigma }_{\varphi }=1/10$ (values that are chosen so that RBF widths are concordant with their spacing; ${\sigma }_{{xy}}$ and ${\sigma }_{\varphi }$ are slightly different due to position being linear while orientation being circular), and $c$ being a scaling constant such that the RBF responses sum to 1. For carrier-based decoding, the complex-valued LFP was demodulated, and its real and imaginary components were fed into a linear model with a softmax output, trained to predict RBF responses. For carrier-free decoding, the complex-valued LFP was projected into a layer whose amplitude was transformed using a softmax output, trained to predict RBF responses. KL divergence was used as the loss function for training. After training, RBF responses were smoothed in orientation and time by a range of values (indicated by $\sigma$ and $\tau$ of Supplementary Fig. 5C/D), and the animal’s position and orientation were estimated to be the center of the maximally activated RBF. The average error between the estimated and true position and orientation were calculated. Using the training data, the orientation smoothing that led to the smallest training error (when $\tau$ = 6.4 seconds) was used to estimate position and orientation for test data. This was done separately for each rat and decoder (carrier-based, carrier-free, and neuron); in practice the choice of orientation smoothing had a small effect (Supplementary Fig. 5C/D right panels). Chance-level position decoding was calculated by calculating the mean distance between the rat’s actual position and noise that was uniformly distributed over the range [0,1]. To evaluate LFP (spike) decoders’ dependency on the number of electrodes (neurons), n channels were randomly chosen over 10 repetitions and used as inputs to the decoder, where n sampled powers of 2 from 8 to 256 (rat 1 had 192 electrodes, so this was the number of channels sampled instead of 256 for rat 1). For rat 2, we noticed particularly poor test performance for the last two folds, especially for carrier-free decoding. We traced this to a distributional shift in the eigenspectrum of the LFP in the last fifth of the session. We therefore discarded 2 folds. Alternatively, carrier-free decoding could be rescued for these folds by normalizing the model’s outputs so that their average response to test data matched their average response to training data.

Within-animal model comparison (Fig. 5C, D): For open field decoding, models were compared separately for each animal. The test accuracies of each of 10 folds were used for each rat as samples for statistical testing. Because training sets overlap across folds, the standard t-test generates artificially low *p*-values. One way of addressing this is to use the correlated t-test^66^.

8 $$t(x)=\frac{\underline{x}}{\sqrt{{\sigma }^{2}\left(\frac{1}{n}+\frac{1}{n-1}\right)}}$$

Where $x$ are the per-fold difference in model accuracies, $\underline{x}$. is their average difference, ${\sigma }^{2}$ is their variance, and $n$ is the number of cross-validation folds. The correlad t-test differs from the original t-test by the addition of the $\frac{1}{n-1}$ term, which effectively reduces the sample size. From this, the *p*-value is computed as:

9 $${p}_{1}=1-{{{\mathscr{T}}}}_{n-1}(t(x))$$

10 $${p}_{2}=2|1-{{{\mathscr{T}}}}_{n-1}(t(x))|$$

Where ${p}_{1}$ and ${p}_{2}$ are the 1- and 2-sided p-values, respectively. We used ${p}_{1}$ to test whether carrier-free is more accurate than carrier-based decoding (Fig. 5), whether error is greater during slow vs fast periods (Extended Fig. 5E), and whether the drop in error is greater for carrier-based than carrier-free decoding (Supplementary Fig. 5E). We used ${p}_{2}$ to test whether carrier-free differed from spike decoding (Fig. 5E).

#### Position-conditioned orientation decoding (Fig. 4C, colored dashed lines)

Because the rat’s preferred orientation depended on its location in the open field, our ability to decode orientation could in principle be an artifact of decoding position. To account for this, we derived a model that estimated the rat’s orientation based only on its estimated position. First, we calculated the joint distribution of position and orientation for each rat by deriving RBFs for position only (10 × 10 × 1 grid) or orientation only (1 × 1 × 10 grid). We took the inner product of these two sets of RBFs to estimate the joint distribution of positions and orientations. Then, we estimated the animal’s orientation as the most likely orientation based on the rat’s estimated position.

Velocity-dependence of position decoding (Supplementary Fig. 5E): For each rat, position was low-pass filtered at 3.2 Hz. Velocities were binned into deciles and the average position decoding error was calculated per decile. Differences between decoders were assessed per velocity (see model comparison above). The relative performance during slow vs fast periods was assessed for each decoder, in each rat, by taking the log-ratio of decoding error between samples falling above vs. below the median of velocity. We used t-tests as described above to determine the probability that carrier-free decoding is more stable than carrier-based decoding between fast vs slow periods (${p}_{1}$) or differs in stability from spike-based decoding (${p}_{2}$).

#### Evaluation of DeepInsight Model (Supplementary Fig. 5F)

DeepInsight^19^ was applied to the LFP data sampled at 1250 Hz. A continuous wavelet transform with frequencies over the range 2 to 625 hz, with two voices per octave, was used to define power at each channel as a function of frequency and time. This resulted in a 3 d tensor, fed into a deep convolutional network whose objective was to predict 4 outputs: x and y positional values (in pixels, ~2.27 cm/pixel), speed (in pixels/s), and head direction (in radians). Position, speed, and head direction use the loss functions Euclidean Loss, MAE, and Cyclical MAE respectively when training DeepInsight. These losses were scaled by factors of 1, 2, and 25 respectively, titrating the impact of each output’s values on the training of the network. Other parameters include window_size (the length of a chunk of data, in samples, defining a single sample of data input to the network) and gap_size (the number of samples dropped at the start and end of the test fold) and average_window (the number of samples averaged to downsample the wavelet-transformed data). These parameters were set to 5000, 2500, and 1250 respectively, based on the default values of the DeepInsight package and adjusting for our sampling rate. The average_window parameter was further increased from the default, and position units were defined in pixels rather than cm, as both of these modifications further improved the network’s performance relative to the default settings.

### LFP simulations

#### Generation of multi-electrode LFPs

A population of place cells received two inputs: 1) the position of an agent traversing a unit-length circular track in one direction, and 2) the theta rhythm, encoded as a complex-valued phasor with an amplitude of 1. The place fields of these cells were spaced uniformly along the track, with Gaussian tuning (${n}_{x}$ in Supplementary Fig. 2, with $a=1$ and $\sigma={e}^{-2}$). The theta-coupling of the neuron’s response ${n}_{\varphi }$ exhibited phase precession at a slope specified by

11 $$m=\frac{-\pi }{\sqrt{2}{e}^{-2}}$$

(see Supplementary Fig. 2). The position- and theta-dependent components ${n}_{x}$ and ${n}_{\varphi }$ were multiplied to generate a complex-valued response for each neuron. These responses were randomly mixed to generate a multi-electrode LFP by multiplying the neural responses with a matrix $A$ of size ${\#\; electrodes}\times {\#\; neurons}$. The real and imaginary components of $A$ were sampled from a uniform distribution over the range $[{\mathrm{0,1}}]$ (sampling a 0-mean complex-valued Gaussian distribution led to weaker signals due to more phase cancellation between neurons). Noise was added to the phase of neurons by sampling from a normal distribution, either independently for each neuron (private noise), or identically for all neurons (shared noise). Figure 2A–E*:* 64 neurons were used (for visualization purposes, using few neurons produced visible phase differences across electrodes), with the agent running a single lap in 1.5 seconds, at a sampling rate of 800 Hz and theta rhythm of 8 Hz. A filter was applied (using Matlab’s gausswin function with window size = 16 electrodes and $\alpha=4$) to the columns of mixing matrix $A$ so that the signal would change smoothly across electrodes, and a lowpass filter (Butterworth filter of order 4 and cutoff frequency ${f}_{L}$) was applied to the noise so that it would change smoothly in time. For the shared noise condition, ${\sigma }_{\epsilon }=1$ and ${f}_{L}=8$ Hz. For the private noise condition, ${\sigma }_{\epsilon }=2$ and ${f}_{L}=4$ Hz. Figure 2F*(decoding):* 1000 neurons were used, sampling position and theta phase uniformly over the range [0,1] and [0,2$\pi$], respectively. Noise was sampled from a normal distribution with ${\sigma }_{\epsilon }=1$. Position was discretized into 25 equally-spaced bins for the purposes of decoding.

#### Decoding of simulated LFPs (Fig. 2F)

The response of the neural population was calculated at each discretized location along the track, turning off the noise as well as the theta input. This response was then multiplied by the mixing matrix ${{\bf{A}}}$ to generate LFP matrix ${{\bf{X}}}$. Demixing matrix ${{\bf{W}}}$ was calculated using the formula

12 $${{\bf{W}}}=\frac{{{\bf{X}}}}{{{{\bf{X}}}}^{T}{{\bf{X}}}+{{\boldsymbol{\Lambda }}}}$$

where

13 $${{\boldsymbol{\Lambda }}}={10}^{-1}\times {{\rm{diag}}}({{\rm{diag}}}({{{\bf{X}}}}^{T}{{\bf{X}}}))$$

The demixing matrix $W$ was multiplied by the demodulated LFP to generate a matrix of 25 pθ time series (one for each location along the track). For each sample, the entry with the pθ with the largest real (absolute) value indicated the estimated location for carrier-based (carrier-free) decoding (Supplementary Fig. 2).

#### Prediction of spike phase in simulated neurons (Fig. 2G)

A population of 1000 neurons was simulated and randomly mixed to generate virtual LFPs at 64 electrodes. From this population, 100 neurons were randomly selected to serve as the “recorded” cells for subsequent spike-phase prediction analyses. The activity of these complex-valued neural responses was predicted using the following models:

1. θ × pos: This model specified that firing phase depended on θ and position. The animal’s position was transformed into a 20-dimensional radial basis representation using raisedcosinebasis.m (available at https://github.com/pillowlab/), and each basis function was multiplied by the θ rhythm, yielding complex-valued regressors that captured position-dependent θ modulation (allowing it to encode phase precession).
2. θ × pos + nrns: This model included the θ × pos regressors along with the complex-valued θ-band activity of the other 99 neurons, allowing for prediction based on shared population activity.
3. All LFPs: This model used the complex-valued θ-band signal from all 64 LFP electrodes as regressors, incorporating spatially distributed information related to θ, position, and shared noise.

Each model produced a predicted complex-valued signal for the target neuron. The neuron’s average activity was then calculated as a function of the predicted phase. Narrower distributions indicated stronger phase concentration and closer alignment between the neuron’s actual and predicted activity.

#### Prediction of spike phase in physiological recordings (Fig. 2H)

Neural signals were preprocessed as described above and spike-phase prediction was performed using the same three regression models described above. For each model, the phase of the predicted complex-valued signal was extracted at the time of each spike, and the resulting distribution was plotted as a histogram. Again, narrower distributions indicated better spike-phase prediction.

### Reporting summary

Further information on research design is available in the Nature Portfolio Reporting Summary linked to this article.

## Supplementary information

Supplementary Information

Reporting Summary

Transparent Peer Review file

## Source data

Source Data

## Data Availability

The data generated in this study have been deposited in the figshare database under accession code 24757638 for 3-arm maze data^67^, and under accession code 26103253 for open field data^68^. The processed data used to generate plots provided in the Supplementary Information/Source Data file. Source data are provided with this paper.

## Code availability

Code is available at (https://github.com/beatLaboratory/TIMBRE).
