# Supplementary material for: Deciphering hippocampal place codes in weak theta rhythms
Source: Nat Commun. 2026 Feb 13;17:2735. doi: 10.1038/s41467-026-69438-5 (PMC13013841; doi:10.1038/s41467-026-69438-5)

Supplementary Fig. 1. Validation of position decoding during stay periods. A) Velocity profile of rats in maze arms and near ports. (i) Velocity profile during a single trial (blue= 'run', red= 'stay') shows transient elevated speed at the beginning and end of time at port. (ii) Histogram and (iii) cumulative distribution of velocities shows a clear separation between run and stay periods. B) Comparison of linear and Bayesian spike decoders. Decoders perform equivalently during Run and Stay periods. n = 4 sessions. **C)** Schematic of alternative models: rather than arising from a place code ("P"), place information in the LFP may reflect theta power, ripple events, movement velocity, or reward-related behaviors (e.g., licking) that co-vary with spatial location ("b,θ"). **D)** Performance of classifiers trained to decode which of two maze arms is occupied, from: LFPs directly (models 1-2), non-place variables (model 3), reconstructions of the LFP using non-place variables (models 4–5), or reconstructions of the LFP using non-place variables sampled within a third arm, so that location was held constant (models 6–7). Dashed line = chance; **p < .01, ***p < .001, n.s. not significant; n=4 sessions. **E)** Logic of model comparisons. If LFPs support position decoding by encoding non-place variables, then mappings trained in a fixed location (models 6/7) should generalize to new arms. Their failure to do so indicates that LFP-based decoding is not explained by non-place variables. The explanations that are consistent (black) or inconsistent (gray) with each model's performance are shown. The only explanation that is consistent with all models is highlighted in red. Source data and statistics are provided as a Source Data file.

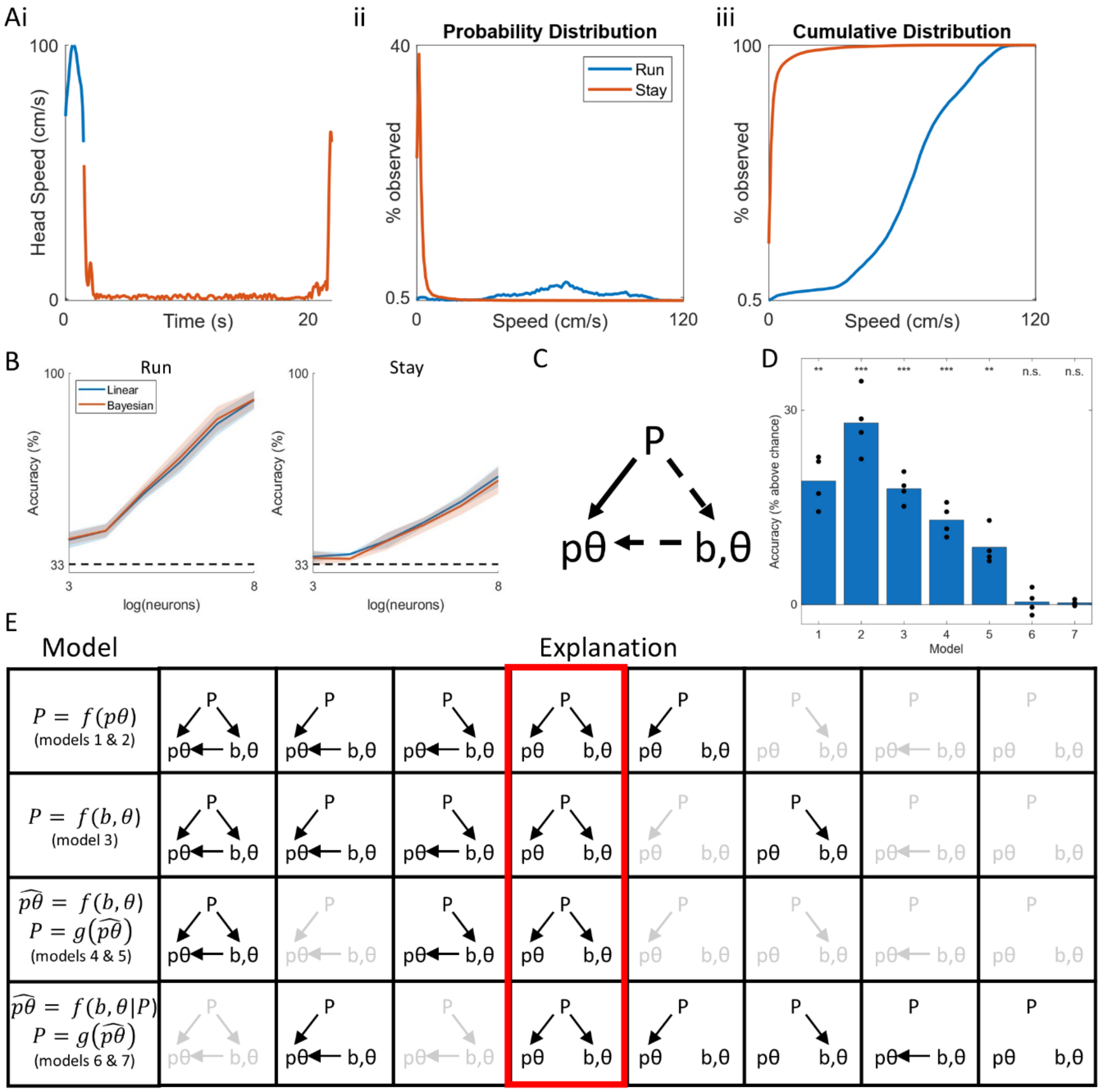

Supplementary Fig. 2. Model of LFP generation. Neurons receive position (**x**(t)) and theta (**θ**(t)) inputs, which modulate position-dependent ($\mathbf{n}_x$(t)) and theta-dependent ($\mathbf{n}_\varphi$(t)) components of activity respectively. Neuronal responses (**n**(t)) of place cells are modeled as a product of the position ($\mathbf{n}_x$(t)) and oscillatory ($\mathbf{n}_\varphi$(t)) components, each of which is complex-valued. Place cell responses are summated using a random mixing matrix A to produce measurements at the electrodes (**LFP**(t)). Multiplying **LFP**(t) by the appropriate demixing matrix **W** results in position-tuned oscillations. For carrier-based decoding, these oscillations are demodulated using **θ**(t), and position is estimated based on the oscillation with the largest real component. For carrier-free decoding, position is estimated based on the oscillation with the largest amplitude.

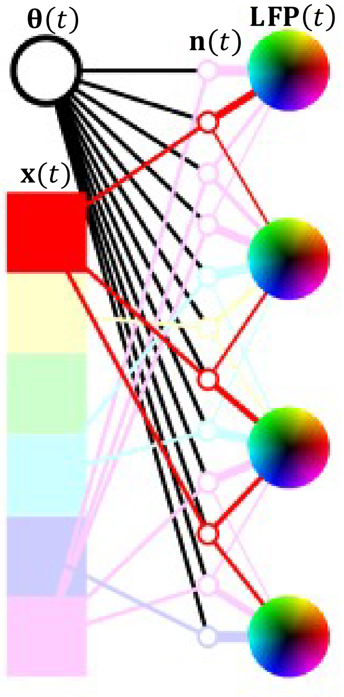

$$\boldsymbol{\theta}(t) = e^{i\boldsymbol{\varphi}(t)}$$

$$\mathbf{n}_\varphi(t) = \boldsymbol{\theta}(t)e^{i((\mathbf{x}(t)-x_0)m+\epsilon}, \epsilon \sim \mathcal{N}(0, \sigma_\epsilon)$$

$$\mathbf{n}_x(t) = ae^{-\frac{(\mathbf{x}(t)-x_0)^2}{2\sigma^2}}$$

$$\mathbf{n}(t) = \mathbf{n}_x(t) * \mathbf{n}_\varphi(t)$$

$$\mathbf{LFP}(t) = \mathbf{A} * \mathbf{n}(t)$$

$$\hat{\mathbf{x}}(t) = \text{argmax}(f(\mathbf{W} * \mathbf{LFP}(t)))$$

where

$$f(\mathbf{z}(t)) = \Re(\mathbf{z}(t) * \overline{\boldsymbol{\theta}(t)}) \quad \text{(Carrier-based decoding)}$$

$$f(\mathbf{z}(t)) = |\mathbf{z}(t)| \quad \text{(Carrier-free decoding)}$$

Supplementary Fig. 3. TIMBRE versus alternatives for identifying informative oscillatory subspaces. A) Pipeline for carrier-free decoding of LFPs. Broadband LFP is bandpass filtered (2-10 Hz) and downsampled and whitened using SVD before being fed into TIMBRE, which learns a collection of oscillations that are informative about behavioral state (in this case, maze arm occupancy). pθ responses are seen in the 4th box from left. **B)** Average response magnitudes of hidden nodes as a function of maze position, for increasing hidden layer sizes. Different colors indicate activations of different hidden nodes. **C)** Feature activations for different versions of TIMBRE (with or without softmax and unit norm), as well as two alternative algorithms, CSP and cICA. In each case, 24 features are depicted. **D)** Comparison of arm classification performance for algorithms shown in C during running (left) and staying (right), mean ± s.e.m., n = 4 sessions from 3 rats. Accuracy is plotted as a function of # learned features per maze arm. Colored asterisks indicate significant differences between TIMBRE and the corresponding decoder (*p<.05, **p<.01, ***p<.001; n=4 sessions). **E)** During running (left), hidden layer activity traces out 1D manifolds corresponding to the linear geometry of each maze arm. Darker shades correspond to earlier phases of the trial. During staying (right), no clear manifold structure is apparent; nonetheless earlier (darker) and later (lighter) phases of the trial appear to segregate. **F)** As with running (Fig. 3D) pθ's activate selectively in one arm; however, there is no clear sequential ordering of pθ activations. **G)** Accuracy of different decoders as a function of time relative to sharp-wave ripple (SWR) onset reveals a momentary dip around the SWR event, mean ± s.e.m., n = 4 sessions from 3 rats. Source data and statistics are provided as Source Data file.

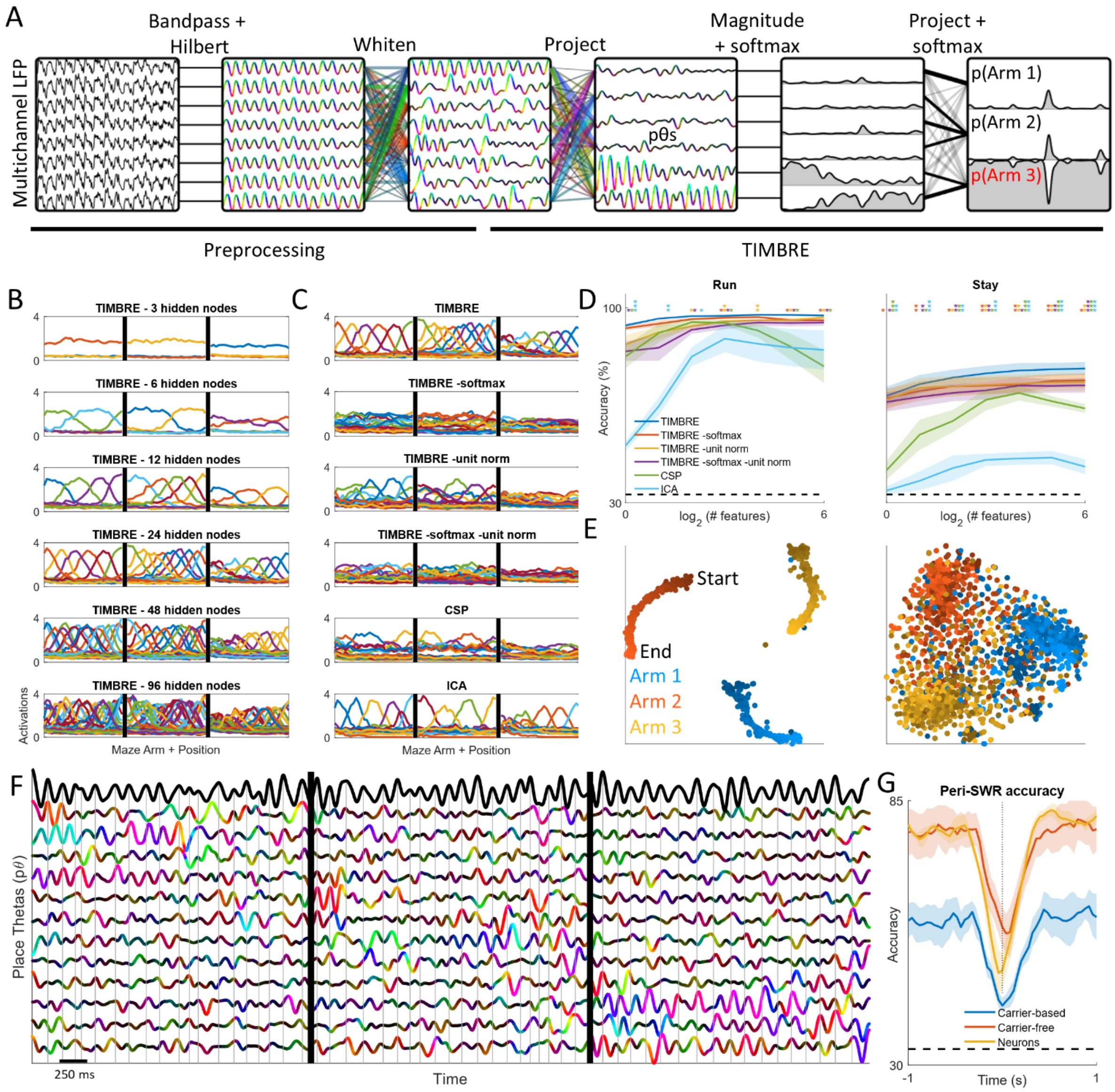

Supplementary Fig. 4 - Defining interneurons and pyramidal cells based on waveform features. **A)** Letters mark properties of action potential waveforms used for clustering. Panel is adapted from *Neuron* 21(1), J Csicsvari, H Hirase, A Czurko, G Buzsáki, "Reliability and state dependence of pyramidal cell-interneuron synapses in the hippocampus", p. 179-189, Copyright (1998), with permission from Elsevier[58]. **B)** Neurons fall into two clusters that are separable by k-means clustering (k=2), identifying putative interneurons (cluster 1) and pyramidal cells (cluster 2). The first two principal components are shown for visualization purposes. **C)** Distributions of each cluster along original feature dimensions show expected differences between interneurons and pyramidal cells. **D)** (left) Distribution of isolation quality for interneurons and pyramidal cells indicates that interneurons are better isolated. (Center and right) Weights of linear model predicting spiking frequency (middle) and θ vs pθ selectivity(right), using only cell type (INT) (magenta) or cell type, log isolation quality (ISO) and log firing rate (FR) (green). Similar weights for the univariate and multivariate regression indicate the dependency on cell type is not explained away by other attributes. Bars indicate 95% confidence intervals for weight estimates. *** $p < .001$, ** $p < .01$, * $p < .05$; n = 834 / 451 neurons for run / stay periods. Source data and statistics are provided as a Source Data file.

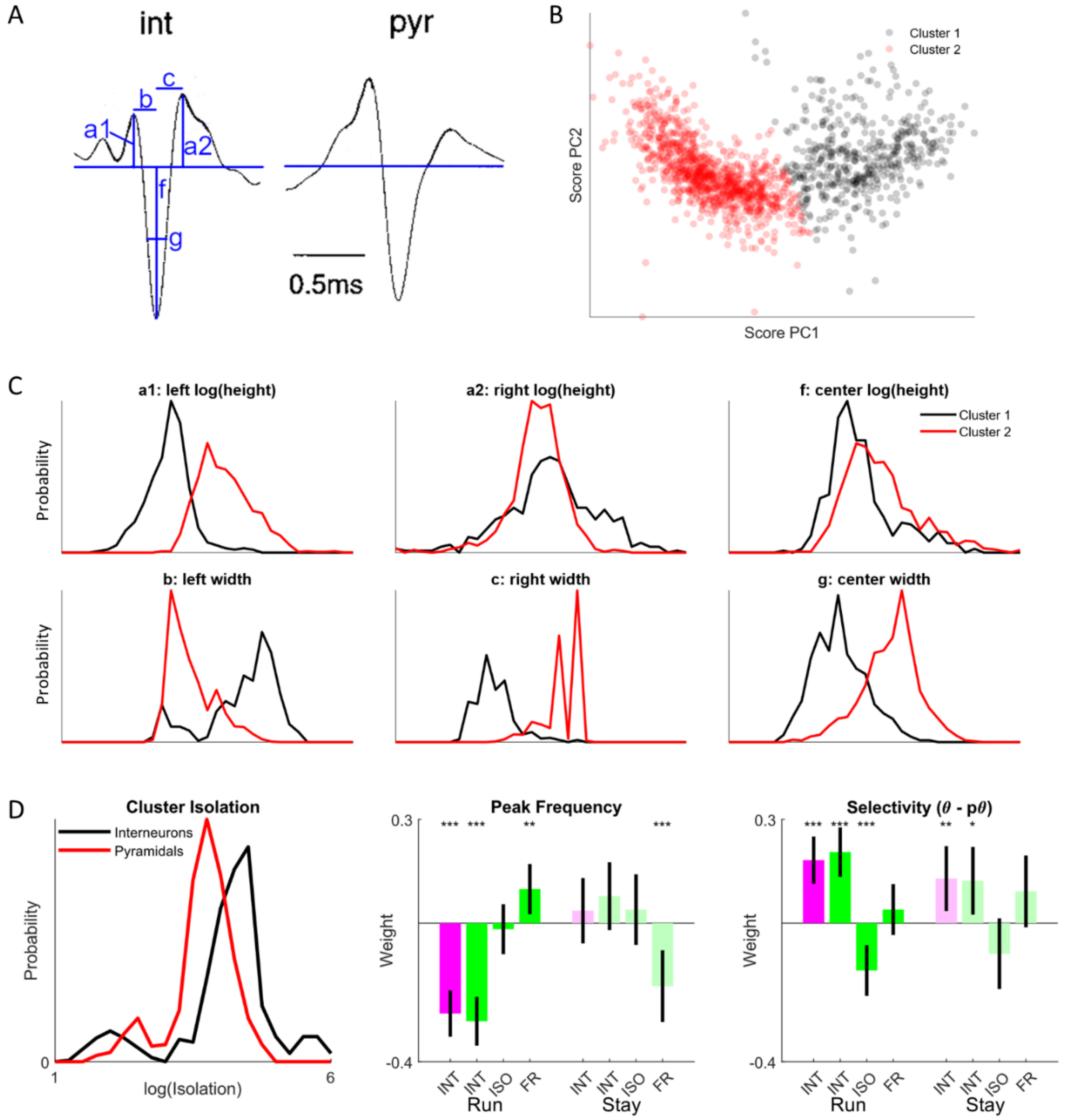

Supplementary Fig. 5 - Influence of model parameters and behavioral state on decoding in the open field. **A)** Feature learning. i) Positions are labeled in a binary manner by splitting the field into two. ii) the hidden layer learns a maximally informative collection of features to distinguish the two halves. **B)** Direct estimation. i) Positions are represented using a collection of radial basis functions (RBFs). RBF widths are depicted here more narrowly than what was used during training, for visual clarity. ii) TIMBRE learns a projection that predicts the activation of each RBF, without a hidden layer. **C)** Effect of velocity on decoder performance. i) θ power changes as a function of velocity. For each rat, data was separated into deciles based on its current velocity, and the average power spectrum of the first principal component of the LFP is shown for each velocity bin. Warmer colors represent higher velocities. Note that this change in θ power and frequency is similar to what is seen during 'run' and 'stay' periods of the maze (**Fig. 1H**). ii) Performance of 3 decoders as a function of velocity. *indicates significant differences between errors for carrier-free vs other decoders at each velocity. iii) Carrier-free decoding showed more stable performance across velocities in both rats than carrier-based decoding. Slow and fast periods are defined as below or above each rat's median speed (*p < .05, **p < .01, ***p < .001; n = 10 (8) folds for rat 1 (2)). **D)** and **E)** For rats 1 and 2 respectively, position (top) and orientation (bottom) error as a function of temporal filter τ (left) and orientation filter σ used to smoothen decoder output. Shaded regions show mean ± s.e.m. of test accuracy for , n = 10 (8) folds for rat 1 (2). For rat 2, two folds were excluded due to distributional shift (see methods). **F)** TIMBRE and DeepInsight[19] show comparable performance for position and orientation decoding. For box plots in Ciii and F, red line = median; box edges = 25th and 75th percentile; + = outliers; whiskers span all data points not marked as outliers. Source data and statistics are provided as a Source Data file.

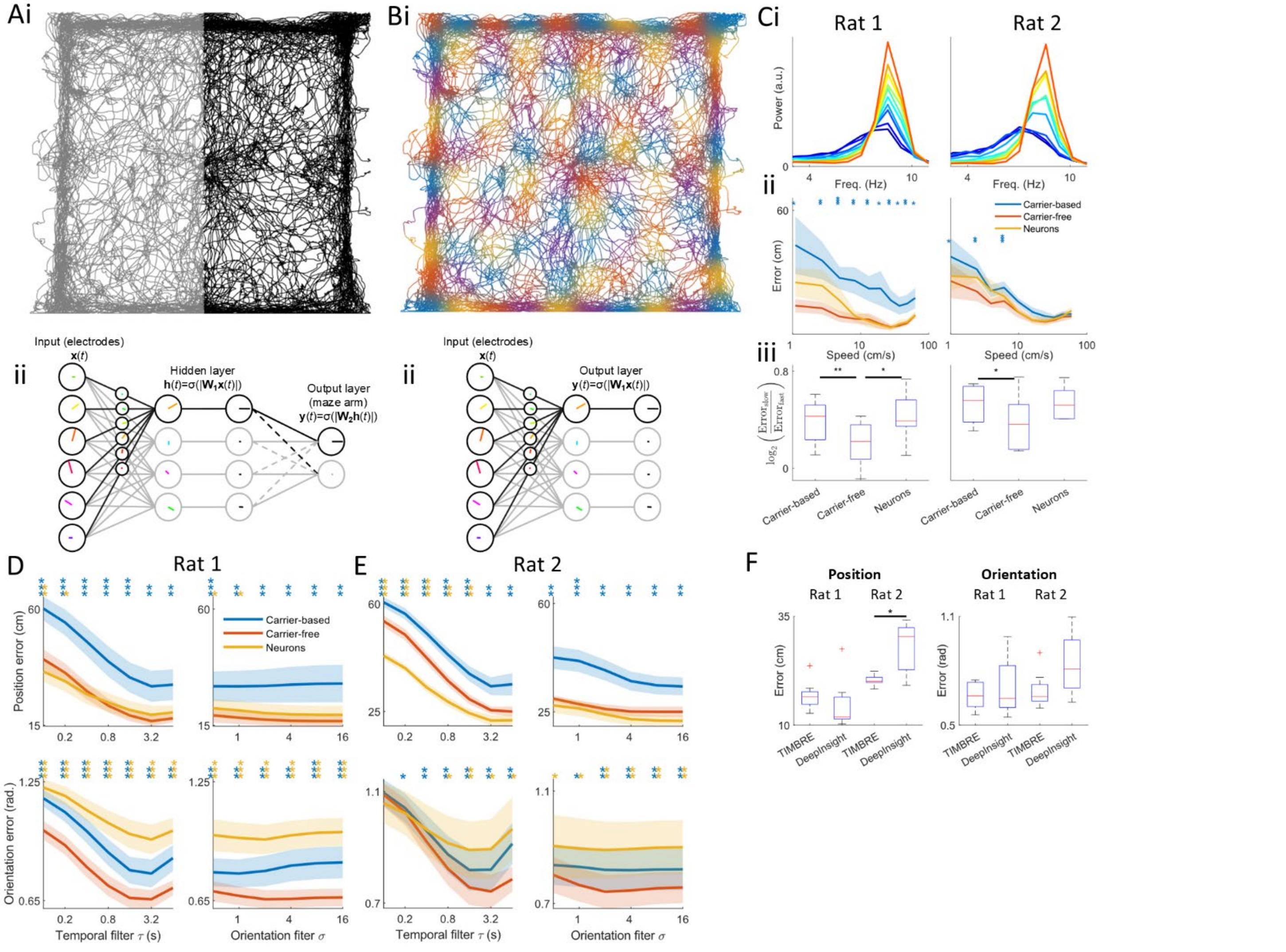

Supplementary Fig. 6 - Proposed relationship between θ and pθ rhythms and hippocampal physiology. The θ rhythm (gray circles), readily visible at individual recording sites, may arise from a population of neurons (black triangles) such as interneurons in the hippocampus and/or the medial septum. Conversely, the pθ rhythms (red circles), extracted by TIMBRE, are spatiotemporal waveforms whose shape is determined by place-tuned cells responding to the current location of the rat in its environment. Cells tuned to different locations are depicted in different colors, with red cells responding to the rat's current location. During periods of running (left, blue box), θ and pθ rhythms are robust and strongly coupled. In this condition one cannot tell whether pθ arises due to a common θ input acting as a pacemaker (black arrows), direct coupling among place-tuned neurons acting as an assembly (red dashed arrows), or both. During stay periods (right, red box) θ is less rhythmic, and the pacemaker and assembly models (H1 and H2, respectively) predict different outcomes. H1 predicts that when θ is weak, place-tuned neurons are unable to synchronize and generate a consistent pθ. H2 predicts that during weak θ, place-tuned cell assemblies can resonate autonomously without θ input (red solid arrows), generating a detectable pθ that is incoherent with θ. While the cells generating θ and pθ are shown here as anatomically segregated, they may be interspersed. Our results are more consistent with H2 and underscore the value of behaviorally informative oscillatory patterns (e.g. pθ) that overlap with more prominent oscillations (e.g. θ) in space, time, and frequency.

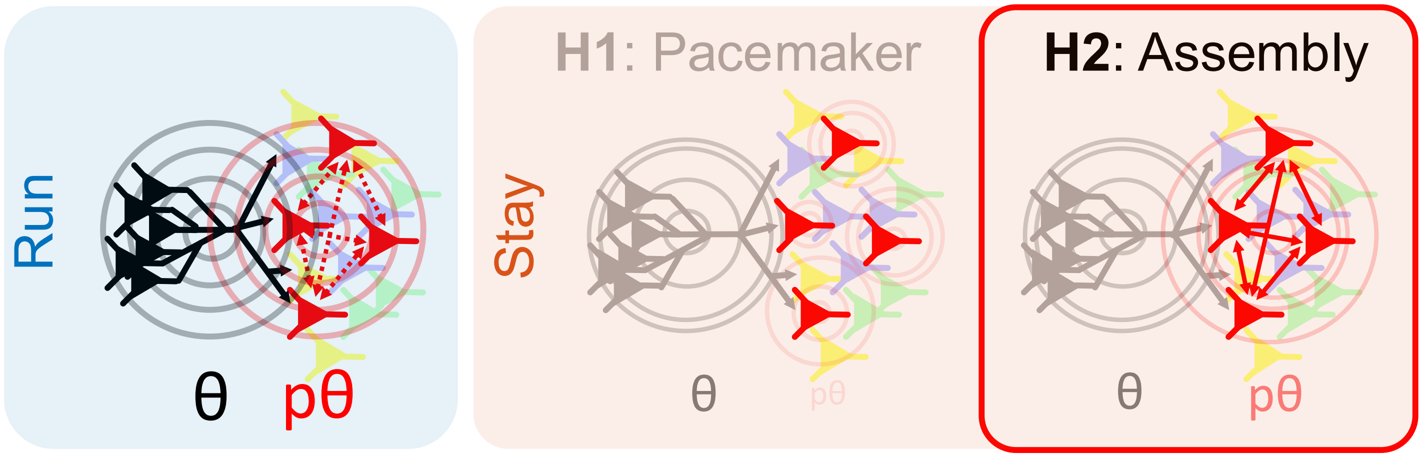

Supplement: Supplementary file 1 — Supplementary Information [file 41467_2026_69438_MOESM1_ESM.pdf]
